# Signatures of adaptive divergence among populations of an avian species of conservation concern

**DOI:** 10.1111/eva.12825

**Published:** 2019-07-09

**Authors:** Shawna J. Zimmerman, Cameron L. Aldridge, Kevin P. Oh, Robert S. Cornman, Sara J. Oyler‐McCance

**Affiliations:** ^1^ Department of Ecosystem Science and Sustainability and Natural Resource Ecology Laboratory, Colorado State University in Cooperation with U.S. Geological Survey Fort Collins Science Center Fort Collins Colorado; ^2^ U.S. Geological Survey Fort Collins Science Center Fort Collins Colorado

**Keywords:** adaptive divergence, local adaptation, sage‐grouse, signature of selection

## Abstract

Understanding the genetic underpinning of adaptive divergence among populations is a key goal of evolutionary biology and conservation. Gunnison sage‐grouse (*Centrocercus minimus*) is a sagebrush obligate species with a constricted range consisting of seven discrete populations, each with distinctly different habitat and climatic conditions. Though geographically close, populations have low levels of natural gene flow resulting in relatively high levels of differentiation. Here, we use 15,033 SNP loci in genomic outlier analyses, genotype–environment association analyses, and gene ontology enrichment tests to examine patterns of putatively adaptive genetic differentiation in an avian species of conservation concern. We found 411 loci within 5 kbp of 289 putative genes associated with biological functions or pathways that were overrepresented in the assemblage of outlier SNPs. The identified gene set was enriched for cytochrome P450 gene family members (CYP4V2, CYP2R1, CYP2C23B, CYP4B1) and could impact metabolism of plant secondary metabolites, a critical challenge for sagebrush obligates. Additionally, the gene set was also enriched with members potentially involved in antiviral response (DEAD box helicase gene family and SETX). Our results provide a first look at local adaption for isolated populations of a single species and suggest adaptive divergence in multiple metabolic and biochemical pathways may be occurring. This information can be useful in managing this species of conservation concern, for example, to identify unique populations to conserve, avoid translocation or release of individuals that may swamp locally adapted genetic diversity, or guide habitat restoration efforts.

## 1 INTRODUCTION

The investigation of adaption in populations and the underlying molecular mechanisms are key topics in ecology, evolutionary biology, and conservation. Groups within a species which can be used to guide management and conservation efforts, termed conservation units (Fraser & Bernatchez, 2001), can be identified through characterization of adaptive divergence. For example, knowledge of adaptive variants in a population could determine which populations can serve as source and recipient for augmentation efforts (Sampson & Byrne, 2016). Additionally, adaptive variation could inform whether augmentation should be done at all (Benedict, Oyler‐McCance, Braun, & Quinn, 2003), guide development of captive breeding programs (Williams & Hoffman, 2009), aid in monitoring and maintaining locally adapted variation in populations, or be used to identify evolutionarily significant units (ESUs; Funk, McKay, Hohenlohe, & Allendorf, 2012). While diversity at putatively neutral genetic markers has long been used to characterize populations, advances in DNA sequencing technology (Mardis, 2008; Metzker, 2010; Shendure & Ji, 2008) and methods to separate neutral and functional genetic variation (Allendorf, Hohenlohe, & Luikart, 2010) have facilitated a shift in focus to understanding the role genetic diversity plays in adaptation to local environments (Nielsen, 2005; Schweizer et al., 2016; Wenzel & Piertney, 2015; De Wit & Palumbi, 2013). Genomic methods can be particularly valuable for characterizing adaptive divergence in species where traditional approaches to evaluate local adaptation (i.e., reciprocal transplant experiments) are not feasible, such as with federally protected species (Funk et al., 2012).

**Figure 1:**
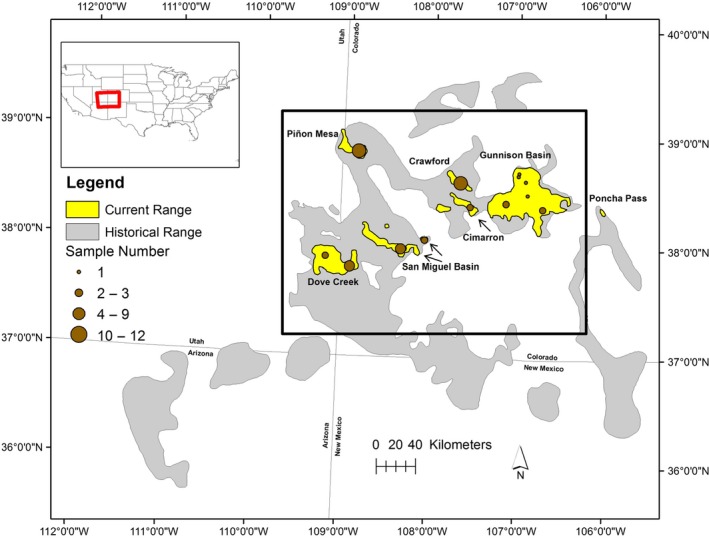
Historical (gray) and current (yellow) distribution of Gunnison sage‐grouse in the southwestern United States. Populations labeled with respective names. Black rectangle designates the study area. The historic range map is as described by Braun et al. (2014); the two northernmost portions of the historic range correspond to an unknown species of sage‐grouse and are not verified by Colorado Parks and Wildlife (Gunnison sage‐grouse Rangewide Steering Committee, 2005). Sample locations are indicated on the map as point of variable size, scale by number of samples collected at the location

The Gunnison sage‐grouse (*Centrocercus minimus*) is a sagebrush (*Artemisia* spp.) obligate avian species persisting as seven isolated populations with low gene flow and high genetic differentiation (Oyler‐McCance, St John, Taylor, Apa, & Quinn, 2005). A single population, the Gunnison Basin, supports the majority of the species (~85%–90% of ~5,000 individuals) with the remaining birds residing in smaller satellite populations (United States Fish & Wildlife Service, 2014). Historically, Gunnison sage‐grouse occurred across ~46,521 km^2^ of sagebrush habitat in Colorado, Utah, New Mexico, and Arizona (Schroeder et al., 2004). Land‐use change in sagebrush habitat has reduced the species to just 8% of the historical range with birds remaining only in southwestern Colorado and southeastern Utah (Figure 1; Braun et al., 2014; Schroeder et al., 2004). In 2014, the species was listed as threatened under the Endangered Species Act (United States Fish & Wildlife Service, 2014). As a sagebrush obligate, Gunnison sage‐grouse requires sagebrush cover for habitat during all life stages (Patterson, 1952; Wallestad & Eng, 1975), and as a source of forage, with up to 99% of winter diet consisting of sagebrush leaves (Braun, Britt, & Wallestad, 1977; Braun, Connelly, & Schroeder, 2005; Young, 1994). Differences in local population environmental conditions also exist (Gunnison sage‐grouse Rangewide Steering Committee, 2005). Each population is centered in a relatively isolated area of the species range and has variable topography and environmental conditions covering a range of average annual precipitation, average annual temperature, and dominant vegetation (Gunnison sage‐grouse Rangewide Steering Committee, 2005; Table 1 and Figure S1.1). Of particular interest to the species and local adaptation are the observed differences in local dominant sagebrush species: Cimarron is dominated by diverse sagebrush cover; Gunnison Basin is dominated by big sagebrush (*Artemisia tridentata* ssp.); Crawford is dominated by big sagebrush and black sagebrush (*A. nova*); Dove Creek has patchy big sagebrush and black sagebrush cover throughout; San Miguel is dominated by lowbrush sage (*A. arbuscula*) at low elevations and more contiguous low, black, and big sagebrush cover at higher elevations; and Piñon Mesa is dominated by big and silver sagebrush (*A. cana*) at lower elevations and patchy big and silver sagebrush at high elevations.

**Table 1:** Environmental characteristics of Gunnison sage‐grouse populations

Population	Pop. Est.	Dom. Veg.	Elev. (m)	PPT (mm)	TMP (°C)	TMAX (°C)	TMIN (°C)	Ann. TMIN (°C)
Cimarron	25	Sagebrush, oakbrush, agriculture	2,133–2743	478.05	5.3	24.32	−11.68	−1.46
Crawford	191	Sagebrush, piñon pine, juniper	1549–2749	512.54	12.4	24.72	−10.52	−0.13
Dove Creek	196	Sagebrush, agriculture	2011–2468	398.29	9.3	26.49	−9.36	0.96
Gunnison Basin	4,763	Sagebrush	2,180–3100	376.61	3.2	22.32	−15.81	−4.53
Piñon Mesa	167	Sagebrush, oakbrush	2,438–2749	486.49	4.9	24.90	−10.22	0.18
San Miguel	334	Sagebrush, low sage	1920–2164	479.18	8.6	25.49	−10.35	−0.22

Note

Pop. Est. = population estimates from 2005 (United States Fish & Wildlife Service, 2014); Dom. Veg. = dominant vegetation cover type (sagebrush = *Artemisia tridentata* sp.; oakbrush = *Quercus gambelii*; piñon pine = *Pinus edulis*; low sage = *Artemisia arbuscula*); Elev. = elevation range of population area (m); PPT = average annual precipitation (mm); TMP = average annual temperature (°C); and to represent the extreme temperatures in each population, TMAX = July maximum temperature (°C), TMIN = January minimum temperature (°C), and Ann. TMIN = annual average minimum temperature (°C).

Although populations are close in proximity (33.34 to 203.72 km apart) relative to observed dispersal capabilities (up to 120–240 km for greater sage‐grouse in mostly contiguous habitat; Cross, Naugle, Carlson, & Schwartz, 2017; Newton et al., 2017; Tack, Naugle, Carlson, & Fargey, 2011), genetic differentiation between populations is relatively high (Oyler‐McCance et al., 2005), suggesting low levels of homogenizing gene flow which might otherwise limit local adaptation. Conversely, some gene flow can increase the local genetic variation in a population and therefore provide more opportunities for natural selection to result in local adaptation (Lenormand, 2002; Whiteley, Fitzpatrick, Funk, & Tallmon, 2015), which suggests observed low levels of gene may promote local adaptation in the different local habitat patches. The male‐dominant polygynous mating system of sage‐grouse skews mating success among males (Wiley, 1973; Young, Braun, Oyler‐McCance, Hupp, & Quinn, 2000) and imposes strong sexual selection which could lead to rapid morphological and/or behavioral changes and further divergence among isolated groups (Ellsworth, Honeycutt, & Silvy, 1995; Oyler‐McCance, St. John, & Quinn, 2010; Spaulding, 2007; Uy & Borgia, 2000). The skew in mating success decreases effective population size (Stiver, Apa, Remington, & Gibson, 2008). This mating skew, along with small population size, also indicates genetic drift could overwhelm the efficacy of selection for local adaptation.

Previous studies have found evidence for significant genetic divergence within some sage‐grouse populations. Isolated populations of greater sage‐grouse (*C. urophasianus*) are genetically distinct enough at neutral loci to warrant consideration for special protection (Benedict et al., 2003; Oh, Aldridge, Forbey, Dadabay, & Oyler‐McCance, 2019). An evaluation of genetic variation at cytochrome P450 genes and additional candidate genes related to metabolism of plant secondary metabolites (PSMs) in greater sage‐grouse identified evidence for positive selection, potentially pointing to local dietary adaptation (Oh et al., 2019). The cytochrome P450 superfamily of genes have broad roles in physiological and toxicological processes (Kubota et al., 2011). Importantly, some of the members of this gene family are involved in metabolism of PSMs (Miyazawa, Shindo, & Shimada, 2001; Skopec, Malenke, Halpert, & Denise Dearing, 2013), like the monoterpenes, sesquiterpene lactones, and phenolics found in sagebrush species (Kelsey, Stephens, & Shafizadeh, 1982). However, taken together with the relevant environmental variation among Gunnison sage‐grouse populations, we became interested in whether there was evidence for adaptive divergence among the populations.

In this study, we examined SNP allele frequencies in six populations along with environmental covariates to address two main research questions about adaptive divergence at the genomic level. First, is there evidence of adaptive divergence among populations of Gunnison sage‐grouse? Second, can we link signals of adaptive divergence to putative gene function? Identification of genes or groups of related genes potentially under adaptive divergence can help elucidate critical factors in the ecology of this threatened species, to be validated and elaborated with further targeted study.

## 2 MATERIAL AND METHODS

### 2.1 Study system

Our study area encompassed the entire species range excluding the eastern most population, Poncha Pass (Figure 1). The Poncha Pass population is thought to have been extirpated in the 1950s and re‐established with Gunnison Basin individuals beginning in the 1970s, persisting only due to ongoing translocation (Nehring & Braun, 2000). For these reasons, the Poncha Pass population was excluded from our analyses.

### 2.2 Genetic samples

Blood samples were collected from 254 birds captured using spotlight trapping methods (Giesen, Schoenberg, & Braun, 1992; Wakkinen, Reese, Connelly, & Fischer, 1992) as part of a previous study (1996–2004; Oyler‐McCance et al., 2005), and DNA was extracted using either a phenol–chloroform method (Kahn et al., 1999) or the Genomic Prep Blood DNA Isolation Kit (Amersham Biosciences) (see Oyler‐McCance et al., 2005, for complete details on collection and DNA extraction). From the 254 samples collected, a subset was chosen for reduced representation sequencing based on population of origin and relatedness. Marker‐based estimates of relatedness (Lynch & Ritland, 1999) were used to select unrelated individuals within each population (see Appendix S2 for a summary of relatedness for selected samples and STRUCTURE analysis). Relatedness estimates for all 254 samples were based on 22 microsatellite genotypes from a previous study (Zimmerman, Aldridge, Apa, & Oyler‐McCance, 2019). The exception was the Cimarron population, for which there were four samples in total; consequently, all Cimarron samples were included. All other populations had 12 samples included in the library preparation.

### 2.3 Library preparation

We accomplished SNP identification using an adapted version of the ddRAD protocol as first described by Peterson, Weber, Kay, Fisher, and Hoekstra (2012). The double digestion utilizes two restriction enzymes which cut the DNA at different frequencies. We used Sau3AI (5,000 units/ml; New England BioLabs) as our common four‐cutter and SPEI (10,000 units/ml; New England BioLabs) as our rare six‐cutter. The digestion reaction for each sample had a total volume of 20 µl: 2 µl T4 10× DNA ligase buffer (New England BioLabs), 0.2 µl bovine serum albumin (BSA; New England BioLabs), 1 µl of each digestion enzyme, 2.8 µl of double‐deionized water, and 13 µl of whole genomic DNA adjusted to a concentration of 77 ng/µl. The digestion was accomplished by incubating all samples at 37°C for 2 hr, then increasing the heat to 65°C for 15 min to kill enzymes, and finally cooling the reaction back to 37°C and holding at temperature. While at 37°C, 1 µl of 10 µM stocks of P1 and P2 (individually barcoded) restriction site‐associated adaptors (Integrated DNA Technologies) was added to each sample and left to equilibrate for 3 min in order to allow adapter dimers to separate. Additionally, the P1/P2 adapter included a degenerate base region to allow identification of PCR duplicates in the bioinformatics stage (Schweyen, Rozenberg, & Leese, 2014). Once the reaction was in equilibrium, 1 µl of T4 ligase (400,000 units/ml; New England BioLabs) was added to each sample. In order for the adapters to ligate to the digested DNA, the temperature was then reduced to 16°C and held for 30 min. The ligase was inactivated through holding the temperature at 65°C for 20 min. The ligation reaction was then diluted with 80 µl of ddH_2_O and then cleaned using 65 µl of SPRI beads (Applied Biological Materials Inc.) to remove adapter dimers present in the reaction. To amplify DNA fragments, we performed a 10 µl PCR using 2 µl of cleaned ligation for each sample, 1 µl 10× Buffer (Fisher Scientific), 1 µl dNTPs, 0.2 µl each of the forward and reverse primers, 0.2 µl AmpliTaq Gold (Fisher Scientific), and 5.4 µl ddH_2_O. The thermocycler protocol for the PCR consisted of 22 cycles of the following: 95°C for 30 s, 55°C for 30 s, and 72°C for 30 s. Each sample was amplified with 9 independent replicates, with all PCR replicates for a sample being pooled into a single sample in an effort to identify and reduce the effects of PCR error. A 16‐µl aliquot of the pooled PCR replicates for each sample was then pooled into a single Eppendorf tube creating a multisample pool, which was then cleaned with SPRI beads in a 1:1 ratio to remove PCR dimers and small‐size amplicons. We performed a final size‐selection step using the Pippen Prep (Sage Science) selecting for fragments between 300 and 500 base pairs. The final size‐selected library was sent to the Genomics and Cell Characterization Core Facility at the University of Oregon in Eugene, Oregon, and was sequenced on the Illumina HiSeq 4,000 platform (Illumina).

### 2.4 Sequence data processing and genotyping

Raw sequencing reads were trimmed at a maximum error probability of 0.05 using CLC Genomics v. 9.5 (Qiagen), allowing at most two ambiguous bases. Reads were mapped to the *Cmin_1.0* Gunnison sage‐grouse draft genome assembly (Oh et al., 2019; GenBank accession: SPOS00000000) with Bowtie2 (Langmead & Salzberg, 2012) using the “very‐sensitive” and “end‐to‐end” parameter sets and filtered on a mapping quality of 20 (Phred‐scaled) with the samtools/bcftools package, v. 1.3 (Li et al., 2009). Potential PCR duplicates were removed by processing unique molecular identifiers with the UMI‐tools package (Smith, Heger, & Sudbery, 2017), using the “unique” identifier detection algorithm.

The samtools/bcftools package was used to merge alignments and identify variant sites in the reference genome. Base composition at sites was computed with the *mpileup* function using the recommended map‐quality adjustment (“‐C” set to 50) and base‐alignment qualities recalculated from the combined data. Indels were called for the purposes of filtering nearby SNP sites (within 3 bp) that could be affected by local misalignment, but were not otherwise used. Genotype likelihoods were estimated with the bcftools *call* function using the multiallelic model, although only biallelic loci were retained. SNP loci were further filtered by requiring a minimum coverage of 960× across all individuals (based on an average of 15X per sequenced individual) and called genotypes for at least 50 of the total 64 individuals. Loci potentially located on sex chromosomes were removed using both coverage and homology information: SNPs on scaffolds putatively homologous with the sex chromosomes of *Gallus gallus* (Oh et al., 2019) were excluded, as were SNPs with unequal coverage in males and females (i.e., if the ratio of male to female mean coverage was outside the range 0.9–1.2). We excluded potentially sex‐linked loci because the proportion of each sex sampled in each population was variable and we wanted to reduce the likelihood of false positives for adaptive divergence due to sampling bias at sex‐linked loci. Sites with low‐frequency minor alleles (below 5%) were also excluded. In addition to filtering variant sites based on these locus‐based criteria, individual genotype calls were removed if coverage was less than 10X for an individual at a given location, regardless of whether a genotype was called by bcftools. After excluding four of the 64 sequenced individuals due to low coverage overall, the final data set included 15,033 loci across 35 “pseudo‐chromosomes” (chromosome scaffolds inferred from synteny with chicken) for 60 individuals (four Cimarron, 12 Crawford, 12 Dove Creek, 12 Gunnison Basin, 10 Pinon Mesa, and 10 San Miguel). Because sample size in Cimarron was low, the power to detect unique outliers in this population was also low. However, inclusion of the Cimarron samples would still help estimate population structure and identify global outliers.

### 2.5 Genetic data analyses

#### 2.5.1 Outlier locus analyses

We identified outlier SNPs using the BayPass core model (Gautier, 2015) and pcadapt (Luu, Bazin, & Blum, 2017) to balance the trade‐off between power and false‐positive rates observed when using parametric (as in BayPass) versus nonparametric (as in pcadapt) outlier analysis approaches. Both approaches perform well under high population structure demographic scenarios, similar to that of Gunnison sage‐grouse (Gautier, 2015; Luu et al., 2017). The core BayPass model expands on the approach implemented in BAYENV (Coop, Witonsky, Di Rienzo, & Pritchard, 2010; Gunther & Coop, 2013) by providing greater computational efficiency, flexibility, and a formal procedure for calculating outlier thresholds. As with BAYENV, this method incorporates a scaled covariance matrix accounting for background population structure which can confound analyses for adaptive variation (Meirmans, 2012). The population covariance matrix was directly estimated with the core model, the Inverse‐Wishart prior set to 1, and both hyperpriors for beta (a.pi, b.pi) set to 1. Five thousand MCMC iterations were performed after discarding a 5,000‐iteration burn‐in and thinning by a factor of 25. Twenty pilot runs of 1,000 MCMC iterations were performed in order to adjust the parameters in the proposal distribution of the Metropolis–Hastings algorithm so that an acceptance rate between 0.25 and 0.40 was achieved. The adjustment parameter (set to 1.25) was used in the pilot runs to adjust the range of possible values from the proposal distributions if the acceptance rate fell outside the desired window. Inference on the core model was through estimates of the XtX statistic. Allele counts were simulated with the *simulate.baypass* R function and the population covariance matrix to generate a pseudo‐observed data set from the core model. Outliers were loci with XtX values exceeding the 99th quantile of the XtX distribution that resulted from the simulated pseudo‐observed data set (false discovery rate (FDR) of 0.01). We verified that the scaled covariance matrix of population allele frequencies estimated from the simulated data was close to the matrix estimated from our data (FMD distance = 0.19, see Gautier, 2015). For pcadapt, we used the pcadapt R package (Luu et al., 2017) with K = 5 based on visual inspection of a scree plot (Figure S3.1). The first 5 principal components generally separate individuals into loose populations (Figure S3.2). We considered loci with a q‐value < 0.01 as outliers.

As an independent evaluation of the SNP data and the ability of BayPass to control for population structure, we compared pairwise *F*_ST_ (as in Weir & Cockerham, 1984) as calculated with the R package diveRsity (Keenan, McGinnity, Cross, Crozier, & Prodohl, 2013) for the neutral SNPs (the SNP data with all outliers removed) to values previously obtained using a microsatellite genotype data set created from the whole available sample set. If SNPs showed the same pattern of differentiation as microsatellite loci, we were confident in the ability of the SNP data and BayPass to estimate population structure (Figure S4).

#### 2.5.2 Genotype–environment association analyses

Environmental covariates tested for association with SNPs were selected based on previously documented effects on sage‐grouse reported in the literature as well as environmental covariates which varied across the species range. Covariates used in model fits included sagebrush cover (Aldridge et al., 2008; Aldridge, Saher, Childers, Stahlnecker, & Bowen, 2012; Baruch‐Mordo et al., 2013; Doherty, Naugle, & Walker, 2010; Harju, Olson, Dzialak, Mudd, & Winstead, 2013; Knick, Hanser, & Preston, 2013; Oyler‐McCance, Burnham, & Braun, 2001), conifer cover and configuration (Baruch‐Mordo et al., 2013; Doherty et al., 2018), dominant shrub type (Aldridge et al., 2008, 2012; Baruch‐Mordo et al., 2013; Doherty et al., 2010; Harju et al., 2013; Knick et al., 2013; Oyler‐McCance et al., 2001), a dryness index (Aldridge & Boyce, 2008), growing degree days (Aldridge & Boyce, 2008), seasonal and annual precipitation (Blomberg, Sedinger, Atamian, & Nonne, 2012), seasonal and annual temperature (Blomberg et al., 2012), seasonal and annual humidity, and phenology metrics derived from NDVI (Aldridge et al., 2012). A total of 72 covariates were initially considered (Table S1.1). We reduced this set to eight variables with a Pearson correlation coefficient <|0.70| for our analyses. This reduced list included spring and fall precipitation, spring maximum temperature, winter vapor pressure deficit (i.e., evapotranspiration), compound topographic index (CTI; a wetness index), green‐up rate, big sagebrush cover, and a dryness index. We also used the loadings of the first three principal components (PCs; PC1 = 37.59%, PC2 = 29.53%, PC3 = 18.85% of the variance) of the eight minimally correlated variables as a covariate in attempt to incorporate multiple covariates in a single model. The principal components analysis was performed with the *prcomp* function in R (see Appendix S1 in Supporting Information for full details on covariates).

Correlation of environmental covariates with SNP genotypes was evaluated using the standard covariate model in BayPass. The model was implemented as in the core model, though including the population covariance matrix estimated with the core model and the addition of regression coefficients which had a uniform prior bounded between −0.3 and 0.3. Covariates with an empirical Bayesian *p*‐value (eBPmc) greater than 4 were considered significantly associated. Gautier (2015) recommends an eBPmc of 3 as a threshold for candidacy; however, we used 4 as a threshold to further control for false discovery. We also used a partial redundancy analysis (RDA), an approach based on a combination of multivariate linear regression and principal components analysis (PCA) that can identify SNPs weakly associated with environmental covariates (Forester, Lasky, Wagner, & Urban, 2018; Rellstab, Gugerli, Eckert, Hancock, & Holderegger, 2015). RDA is a nonparametric approach to identifying candidate adaptive loci which has high power and low false‐positive rates, yet has not been evaluated under a high population structure demographic scenario as observed in Gunnison sage‐grouse. Further, evaluation of a partial RDA (accounting for demographic structure) on a low‐structure system resulted in reduced power and increased false‐positive rates (Forester et al., 2018). However, results of a recent application of a partial RDA to a high‐structure system (global *F*_ST_ = 0.48; Brauer, Hammer, & Beheregaray, 2016) were consistent with an independent transcriptomics study (Brauer, Unmack, & Beheregaray, 2017), suggesting the partial RDA performs well in high‐structure systems. We therefore report the results of a partial RDA as a complement to the BayPass standard covariate model. For the partial RDA, the genetic data were recoded to a 0,1,2 format, where individuals heterozygous at a locus were designated as 1, and homozygotes were designated as 0 and 2 for the reference and alternate alleles, respectively. We accomplished the partial RDA by calculating a genetic distance matrix (*vegdist* function using the “bray” method in the vegan R package; Oksanen et al., 2017) and identifying the significant genetic axes of a principal coordinates analysis on the genetic distance matrix (pcoa function in the ape R package; Paradis & Schlier, 2018); significance of genetic axes was based on the broken‐stick criterion (Legendre & Legendre, 2012). We then created spatial predictors by calculating Moran eigenvector maps (MEM; based on a Gabriel graph neighborhood and inverse distance weights) in the R packages spdep (Bivand & Piras, 2015) and adespatial (Dray et al., 2019). We used the first 4 genetic axes (as determined by the broken‐stick criterion), as the response variable in a forward selection algorithm including all MEMs (*rda* and *ordistep* functions in the vegan R package; Oksanen et al., 2017), which stops adding variables when the adjusted R^2^ of the full model is exceeded, and a significance threshold for inclusion of *P*_IN_ = 0.01. Significant MEMs that were not correlated with environmental covariates at *r*>|0.70| were retained. Environmental covariates were evaluated for correlation and checked for multicollinearity, retaining only a single variable correlated at Pearson's *r *> |0.70| and removing variables with VIF > 10 (Kutner, Nachsheim, & Netter, 2004). Our final partial RDA included only the spring precipitation, fall precipitation, CTI, and proportion of big sagebrush variables after accounting for correlation and multicollinearity. Formal significance of the full model and marginal significance for which constrained axes to be evaluated for candidate loci was performed with the *anova.cca* function and 999 permutations in the vegan package. Significant (*p*‐value < 0.05) marginal constrained axes were retained for evaluation of candidate SNPs (see Figure S5 for corresponding scree plot). Loci in the tails of the distribution of the SNP loadings on each axis were considered outliers. In attempt to keep false positives low, we used a two‐tailed *p*‐value of 0.0027 (based on 3 standard deviations) as a cut off for candidacy.

#### 2.5.3 Linkage disequilibrium and gene ontology enrichment analyses

We phased our SNPs and estimated linkage disequilibrium (LD) decay to determine at what distance candidate loci could be considered physically linked to a putative gene region on the reference genome based on the position of SNPs on pseudo‐chromosomes. To phase our SNPs we used BEAGLE 5.0, setting *N*_E_ to 1,000 as recommended to indicate our data are from a small and inbred population (Browning & Browning, 2007). With the phased SNPs, we calculated LD in vcftools (‐hap‐r2 command) at multiple distances, from SNPs 10 bp to 1Mbp apart. We considered SNPs at the distance where LD as measured by *r* ^2^~0.10 to be physically linked.

We then further investigated the relationships between putative gene products using a gene ontology (GO) enrichment analysis. We used Gowinda v1.12 (Kofler & Schlötterer, 2012) to evaluate overrepresentation of GO terms in individual candidate SNP lists, or lists of loci identified by core or standard covariate models in BayPass, pcadapt, and the partial RDA. Gowinda input includes a list of all the SNPs considered in the outlier analysis, a list of identified candidate loci, a GO association file (FuncAssociate; Berriz, King, Bryant, Sander, & Roth, 2003), and a draft genome annotation file (chicken homology based gene predictions and annotations aligned to the Gunnison sage‐grouse draft genome; Oh et al., 2019). The *p*‐value (before FDR adjusted) is calculated as the proportion of simulations with more genes for a category with at least one candidate locus than the whole observed data set. We used the SNP mode (a gene region containing multiple SNPs was counted once for each SNP and assumed complete linkage equilibrium) to test against the GO categories, with 100,000 simulations to generate the null distribution. We also used gene lists derived from Gowinda for all analyses (BayPass, pcadapt, RDA) to evaluate significantly overrepresented functional annotation terms in DAVID (Database for Annotation, Visualization and Integrated Discovery; Huang et al., 2007) using default parameters. Lastly, we evaluated the potential effect of the candidate SNP variants identified by all tests with SnpEff (Cingolani et al., 2012).

To visualize clustering of individuals into potentially adaptively divergent groups, we performed PCA on the candidate SNP loci and SNP loci in identified gene families with the *princomp* function in R and plotted the first three principal components using ggplot2 R package (Wickham, 2009). For comparison, we also included plots of the first three principal components for analyses on all SNPs and putatively neutral SNPs.

## 3 RESULTS

### 3.1 Population genetic structure check

**Figure 2:**
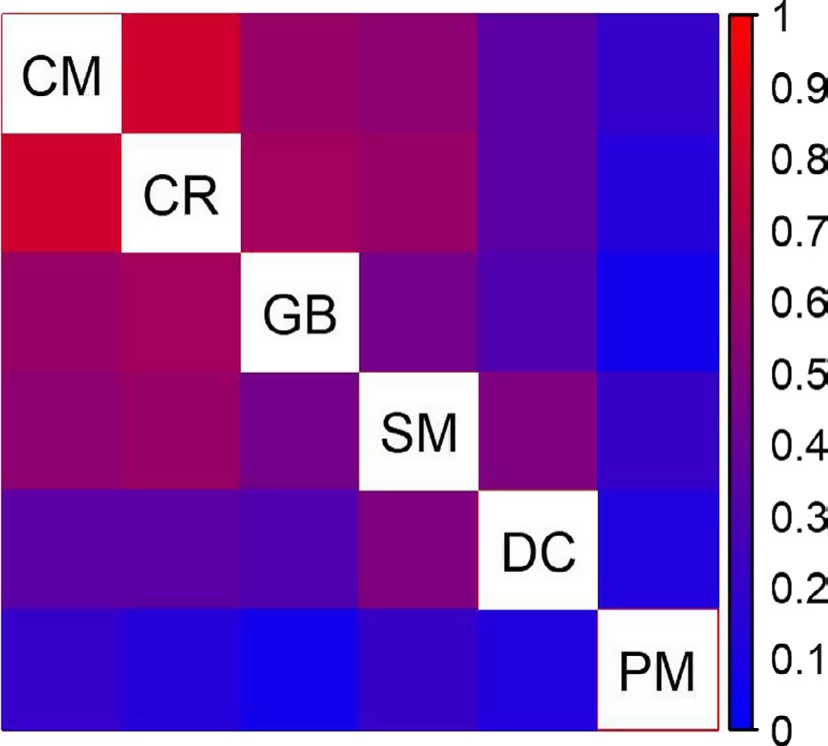
Heat map for the correlation between populations of Gunnison sage‐grouse (low correlation = blue; high correlation = red) derived from the allele frequency covariance matrix used in the BayPass program to account for demographic structure. Population name abbreviations: CM = Cimarron, CR = Crawford, DC = Dove Creek, GB = Gunnison Basin, PM = Piñon Mesa, SM = San Miguel

Pairwise multilocus F_ST_ values as calculated from only putatively neutral SNPs corresponded well by rank with previous microsatellite estimates, although the latter appear to have consistently lower means (Figure S4). The population covariance matrix inferred from neutral SNPs also confirms our previous understanding of population structure. We have included a heat‐plot of the correlation matrix derived from the allele frequency covariance matrix estimated in the BayPass program to illustrate how populations are related (Figure 2).

### 3.2 Genome scans for adaptive divergence and association with environmental variables

**Figure 3:**
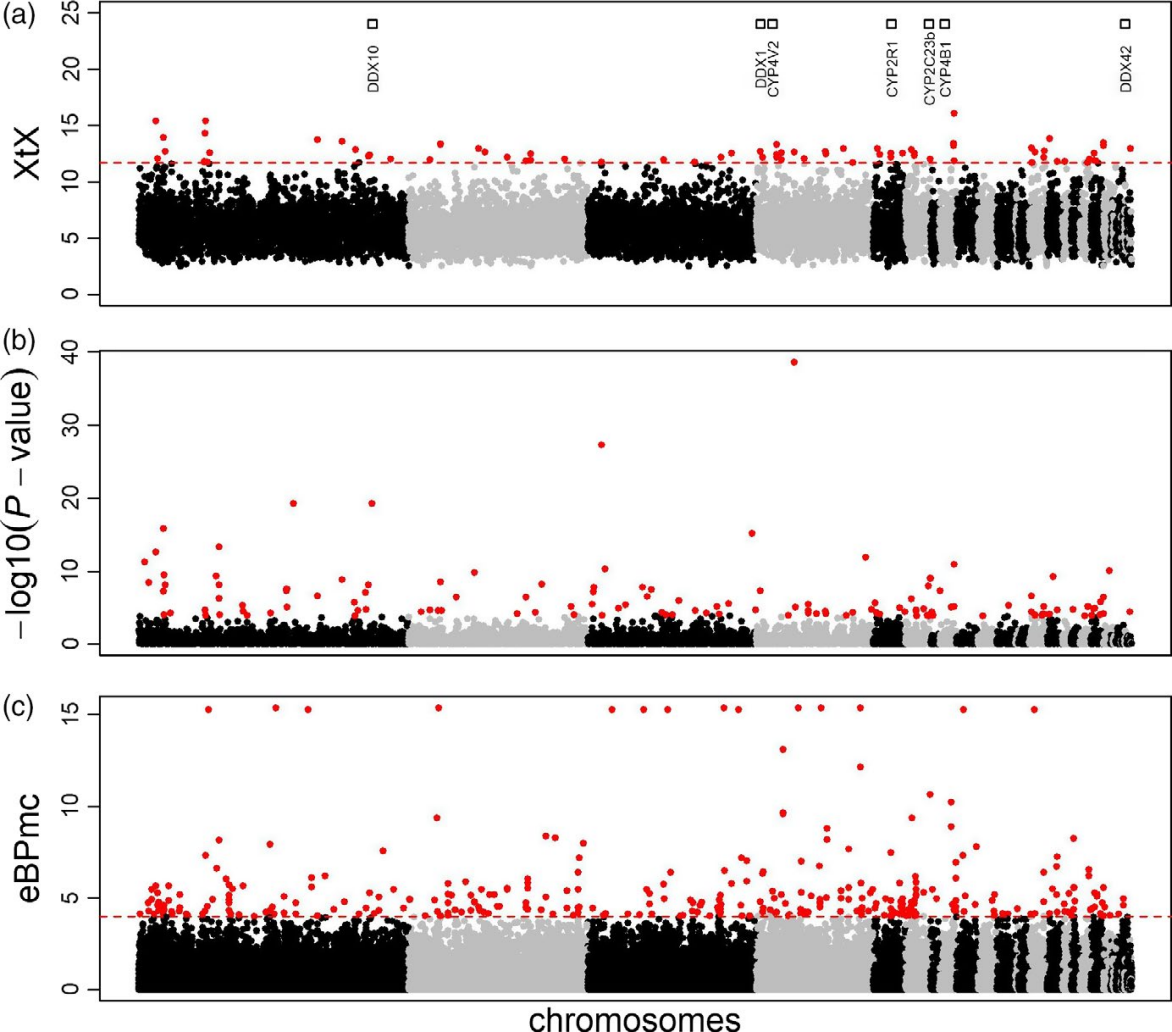
(a) XtX from the core BayPass model, (b) –log10(*p*‐value) from pcadapt, and (c) empirical Bayesian *p*‐value (eBPmc) for each locus or each locus–covariate pair. X‐axis corresponds to the SNP position along pseudo‐chromosomes, alternating gray and black indicate SNPs observed on different pseudo‐chromosomes for Gunnison sage‐grouse. SNPs with XtX or –log10(*p*‐value)> FDR 0.01 or eBPmc > 4 are red. Squares along the top of plot (a) indicate the locations of enriched genes of interest

The BayPass core model identified 76 outlier loci located on 13 of the 35 pseudo‐chromosomes that had SNPs, and pcadapt identified 157 outlier loci located on 17 pseudo‐chromosomes (Table 2, Figure 3a,b). The standard covariate model identified significant associations for all covariates evaluated: 32 SNPs on 8 pseudo‐chromosomes with spring precipitation, 26 SNPs on 8 pseudo‐chromosomes with fall precipitation, 15 SNPs on 7 pseudo‐chromosomes with spring maximum temperature, 37 SNPs on 12 pseudo‐chromosomes with CTI, 30 SNPs on 7 pseudo‐chromosomes with green‐up rate, 36 SNPs on 8 pseudo‐chromosomes with big sagebrush cover, and 45 SNPs on 15 pseudo‐chromosomes with dryness index (Table 2, Figure 3c). Similarly, significant relationships were found with the principal components included as covariates: 40 for PC1 (highest loadings: maximum temperature [0.53], big sagebrush cover [0.54], green‐up rate [−0.53]), 12 for PC2 (highest loadings: spring precipitation [0.53], winter vapor pressure deficit [0.54] and dryness index [−0.5]), and 43 for PC3 (highest loading: CTI [0.76]). See Table S1.3 for all covariate loadings onto PCs. The partial RDA was globally significant (*p* = 0.001) and accounted for 31.2% of the total variation. A total of 602 SNPs were identified as outliers in the tails of the first 4 axes (all with *p* < 0.03). The significant axes accounted for descending amounts of variation (RDA1 = 9.9%, RDA2 = 7.2%, RDA3 = 4.3%, RDA4 = 1.2%) and different numbers of candidate loci (RDA1 = 91, RDA2 = 178, RDA3 = 175, RDA4 = 163). Predictor covariates (highest loading) corresponded to proportion of big sagebrush cover (10 SNPs), spring precipitation (180 SNPs), fall precipitation (212 SNPs), and CTI (200 SNPs). Each of the axes generally displayed an environmental gradient of predictor variables: RDA1 corresponded to an environmental gradient of fall precipitation, RDA2 to a spring precipitation gradient, RDA3 to a CTI and fall precipitation gradient, and RDA4 to a spring and fall precipitation gradient. Plots of the first 4 RDA axes are included in the Appendix (Figure S6). Overlap of loci identified with each analysis varied; no two analyses identified identical lists (Table S7).

**Table 2:** Summary of the number of SNPs (No. Cand. SNPs) showing signatures of adaptive divergence for Gunnison sage‐grouse in different models (Method), the number of chromosomes with candidate SNPs (No. Chrome. W/Cand. SNPs) at FDR 0.01. The number of GO terms associated with each candidate SNP list (No. Sig. GO Terms) and number of unique genes associate with GO terms (No. Genes Assoc. W/GO Terms) at FDR 0.05 and FDR 0.01 are included in the last four columns

Method	No. Cand. SNPs	No. Chrome. W/Cand. SNPs	No. Sig. GO Terms		No. Genes Assoc. W/GO Terms	
Variable			FDR 0.05	FDR 0.01	FDR 0.05	FDR 0.01
pcadapt	156	17	161	0	15	0
BayPass						
‐‐	76	13	51	33	8	2
PC1	40	14	41	11	4	3
PC2	12	4	0	0	0	0
PC3	43	15	0	0	0	0
Spring Precip.	32	8	0	0	0	0
Fall Precip.	26	8	2	2	1	1
Spring Max. Temp.	15	7	0	0	0	0
Winter. Max. Vapor.	27	8	2	2	1	1
CTI	37	12	0	0	0	0
Green‐up Rate	30	7	0	0	0	0
Big Sagebrush	36	8	24	20	1	1
Dryness Index	45	15	55	45	3	2
Partial RDA	602	25	53	22	22	12

### 3.3 Gene ontology enrichment analyses

Eight unique genes from gene sets associated with 51 GO terms (FDR 0.05) were identified in a gene ontology enrichment analysis of outliers identified by the BayPass core model and 15 unique genes from gene sets associated with 161 GO terms (FDR 0.05) were identified with outliers from pcadapt (Table 2). Outlier lists for covariates identified variable numbers of enriched GO terms and associated gene sets, ranging from zero GO terms at FDR 0.05 for PC2, PC3, spring precipitation, spring maximum temperature, CTI, and green‐up rate, to 55 GO terms and three unique gene set genes at FDR 0.05 (45 and two respectively at FDR 0.01) for the dryness index (see Table 2 for summarization of all tests).

Global LD was estimated to be 0.02, and dropped to *r* ^2^ < 0.1 at ~ 350 kbp (Figure S8), a distance much greater than the default distance we used to align candidate SNPS to gene regions (5 kbp). There are likely many genes within the 350 kbp LD blocks, any of which could be the true target of selection. Restricting the distance between candidate SNPs and gene regions increased our confidence that the association between candidate SNP and gene was nonrandom although it ensures that many potential targets of selection would not be included. Of the 950 total candidate SNPs, 411 were located within 5 kbp of one of 289 putative gene regions, and considered in linkage. The majority of candidate SNPs within 5 kbp of a putative gene region were identified as potential modifiers (453) or low impact variants (4) and predicted to result in a synonymous amino acid substitution (2), or were located in introns (260; gene regions excised before translation into proteins), upstream of a coding region (56), or downstream of a coding region (59) by SnpEff (Table 3 and Table S10). Additionally, three SNPs in three putative genes were indicated as nonsynonymous variants and moderate or high impacts to putative gene function (Table S10): protein kinase, DNA‐activated, catalytic polypeptide (PRKDC), mortality factor 4‐like 1 (MORF4L1), and zinc finger and BTB domain containing 2 (ZBTB2).

**Table 3:** Summary of the outlier loci from Gunnison sage‐grouse populations in enriched families or proteins. Findings from pcadapt, the core BayPass model (“core”), the standard BayPass model including principal component 1 (“PC1”), spring maximum temperature (“Spring Tmax.”), green‐up rate, dryness index, and RDA with associated predictor variable (“RDA: Spring PPT,” “RDA: Fall PPT.,” “RDA: CTI”). The gene code is listed in the left‐hand column (see Table S9 for a list of the corresponding full gene names and Table S10 for all putative adaptive genes) followed by the pseudo‐chromosome number where it is located (“Chromosome”), the total number of SNPs identified as outliers in each gene region (“# SNPs”), indication of significance at FDR 0.05 (*) for BayPass in each model. Impact of each SNP as predicted by SnpEff is indicated in the by counts of SNPs in gene region in the corresponding “Effect” column

Gene Code	Chromosome	No. SNPs	pcadapt	core	PC1	Green‐up rate	Dryness index	RDA: Spring PPT	RDA: Fall PPT	RDA: CTI	MODIFIER	Intron	Intergenic	Downstream	Upstream
			Test								Effect				
**Cytochrome P450**															
CYP2C23b	6	3	*							*	3	2	1		
CYP2R1	5	3		*			*		*		3	3			
CYP4B1	8	1							*		1	1			
CYP4V2	4	1				*					2		1	1	
**DEAD Box Helicase**															
DDX1	4	1			*					*	1	1			
DDX10	1	1						*			1			1	
DDX42	27	2							*		4		2		2

Enrichment analysis using DAVID identified several significant GO terms in each database category though none were significant after adjustment for multiple testing, suggesting interesting though potentially spurious relationships. There were four functional enrichment annotation clusters with a top GO term category with *p* < 0.05, which included MAM domain (cluster 1 enrichment score: 1.42), ANK sequence repeat (cluster 2 enrichment score: 1.14), cytochrome P450, E‐class, group 1 (cluster 3 enrichment score: 1.08), and short sequence motif: DEAD box (cluster 4 enrichment score: 0.90) (Table 4; see Table S11 for a complete list).

**Table 4:** Summary of the top 3 enriched GO terms (“Term”) in the top functional annotation clusters for Gunnison sage‐grouse from DAVID that had a term significant at *p* < 0.05. *p* = *p*‐value; B = Benjamini–Hochberg correction; ES = enrichment score. Category (C): OG = orthologous groups; P = proteins; BP = biological processes; SEQ = sequence feature; MF = molecular function. See Table S11 for complete list of functional annotation clusters and corresponding terms. See Table S9 for gene names corresponding to gene codes included in table below

Cluster (ES)	Category	Term	No.	*p*	B	Genes
1 (1.42)	P	IPR000998:MAM domain	3	0.03	1.00	C10ORF112, PTPRT, MEP1A
	P	SM00137:MAM	3	0.03	0.99	C10ORF112, PTPRT, MEP1A
	P	IPR013320:Concanavalin A‐like lectin/glucanase, subgroup	7	0.05	1.00	C10ORF112, PTPRT, SPRYD7, DDX1, LGALSL, MEP1A, LAMA5
2 (1.14)	SEQ	repeat:ANK 5	3	0.02	0.70	PPP1R12A, ANKRD10, ANKRD44
	SEQ	repeat:ANK 4	3	0.02	0.66	PPP1R12A, ANKRD10, ANKRD44
	SEQ	repeat:ANK 2	3	0.04	0.74	PPP1R12A, ANKRD10, ANKRD44
3 (1.08)	P	IPR002401:Cytochrome P450, E‐class, group I	4	0.02	1.00	CYP4B1, CYP2C23b, CYP4V2, CYP2R1
	MT	GO:0005506 ~ iron ion binding	7	0.02	0.97	CYP4B1, CYP2C23b, CYP4V2, FTL, ALOX5, TH, CYP2R1
	P	IPR001128:Cytochrome P450	4	0.03	1.00	CYP4B1, CYP2C23b, CYP4V2, CYP2R1
4 (0.90)	SEQ	short sequence motif:DEAD box	3	0.01	0.58	DDX10, DDX1, DDX42
	BP	GO:0010501 ~ RNA secondary structure unwinding	4	0.03	0.97	AGO2, DDX10, DDX1, DDX42
	SEQ	domain:Helicase C‐terminal	3	0.03	0.75	DDX10, DDX1, DDX42

**Figure 4:**
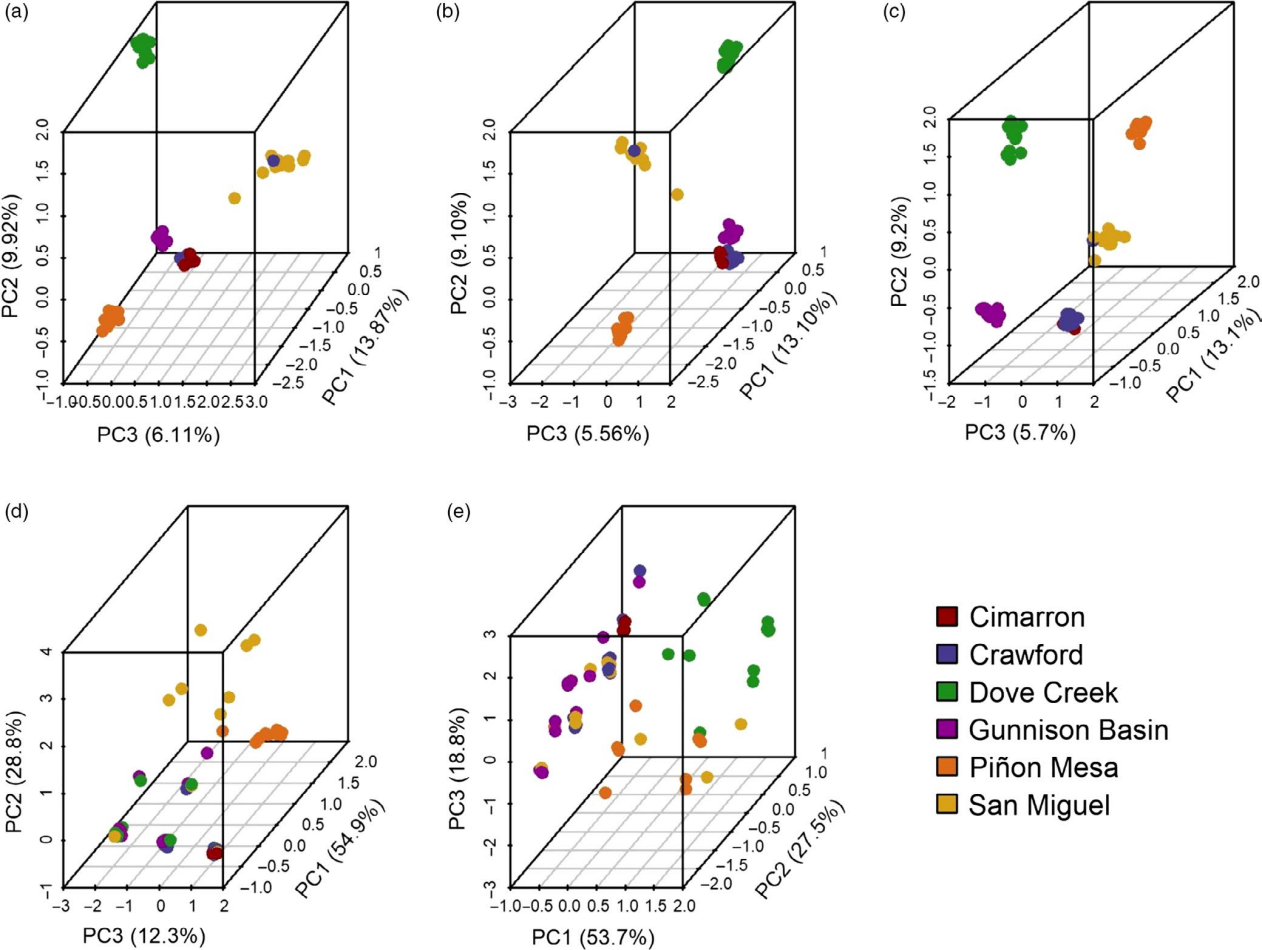
Clustering of individual Gunnison sage‐grouse using 3‐D PCA plots with (a) all SNPs (15,033 loci; first 3 PCs account for 29.9% of the variation in the genotypes), (b) putatively neutral SNPs (14,091 loci; first 3 PCs account for 30.4% of the variation in the genotypes), (c) all candidate SNPs (942 loci; first 3 PCs account for 41.3% of the variation in the genotypes), (d) all cytochrome P450 candidate SNPs (9 loci; first 3 PCs account for 88.1% of the variation in the genotypes), (e) all DEAD box helicase candidate SNPs (4 loci; first 3 PCs account for 100% of the variation in the genotypes—note the different order of the axes). Each point represents an individual color coded by the population where the sample was collected: CM = Cimarron; CR = Crawford; DC = Dove Creek; GB = Gunnison Basin; PM = Piñon Mesa; and SM = San Miguel

Individuals generally clustered by population when candidate loci were used in a PCA (Figure 4c) which was somewhat similar to the clustering of individuals with all and putatively neutral SNP loci (Figure 4a,b), although Crawford and Cimarron cluster more tightly together while Gunnison Basin, Dove Creek, Piñon Mesa, and San Miguel populations appear to separate from the other three populations and each other with candidate loci. PCA plots with SNPs from individual analyses generally showed similar clustering patterns to that of the overall outlier clustering (see Figure S12). A PCA plot of the SNPs within 5 kbp of cytochrome P450 genes showed that most individuals loosely clustered while some San Miguel individuals and nearly all Piñon Mesa individuals clustered away from the remaining individuals (Figure 4d). Similarly, a PCA plot for the loci associated with the DEAD box helicase genes showed very loose clustering, with Dove Creek appearing separate, Piñon Mesa and San Miguel largely clustering together, and Cimarron, Crawford, and Gunnison Basin clustering (Figure 4e).

## 4 DISCUSSION

We found allelic differentiation consistent with adaptive divergence at SNPs associated with potentially important gene families for local ecological adaptation between isolated populations of a single avian species. Additionally, the gene set identified by candidate SNPs was enriched for an ecologically significant gene family for sage‐grouse, the cytochrome P450 gene family. Previously detected genome‐wide resequencing analyses support the signals of divergence associated in this gene family across population in both species of sage‐grouse (Oh et al., 2019). Future work could confirm F_ST_ of outliers in a larger sample of individuals and/or with whole‐genome resequencing within the Gunnison sage‐grouse populations, sequence haplotype blocks in the vicinity of outliers, measure expression of putatively adaptive genes as a function of SNP genotype, and/or evaluate the role of cytochrome P450 genes in chicken models of response to plant secondary compounds. Our findings provide an initial look for genome‐wide signals of adaptive divergence among populations for the Gunnison sage‐grouse.

Identification of signals of adaptive divergence in Gunnison sage‐grouse populations also provides more evidence of natural selection occurring in unexpected situations. First, effective population size can influence the balance between selection and genetic drift. Large effective population sizes are less influenced by genetic drift and therefore natural selection is expected to be more efficient (Frankham, 1996; Gossmann, Keightley, & Eyre‐Walker, 2012). The mating system of Gunnison sage‐grouse indicates the species generally has a small effective population size, and so this work adds to the few documented examples where locally adapted variation persists despite small effective population size (McKay et al., 2001; Phifer‐Rixey et al., 2012). Second, geographic scale also plays a role in the likelihood of divergence. At large geographic scales gene flow is expected to be low among populations allowing divergence to occur even in the absence of strong selection (Rousset, 1997; Slatkin, 1993). At the microgeographic scale (when geographic distances between populations are within the known physical dispersal range of an organism), high gene flow is expected to impede local adaptation (Slatkin, 1987), although some argue microgeographic local adaptation is more common than previously appreciated (Richardson, Brady, Wang, & Spear, 2016). Though not a perfect system to evaluate microgeographic local adaptation, the populations of Gunnison sage‐grouse are located within their known physical dispersal range. Few examples of microgeographic adaptation have been identified in birds, presumably because birds are considered vagile (Charmantier, Doutrelant, Dubuc‐Messier, Fargevieille, & Szulkin, 2016; Langin, Sillett, Morrison, & Ghalambor, 2017; Manthey & Moyle, 2015; Termignoni‐García et al., 2017). Identification of signals of adaptive divergence in Gunnison sage‐grouse populations indicates distinct selective environments (Karlin & McGregor, 1972; Levene, 1953; Urban et al., 2017), a physical limit to dispersal (Fischer & Lindenmayer, 2007; Slatkin, 1987), mating signal divergence (e.g., Langin et al., 2015; Langin et al., 2017), or that any type of assortative mating may be facilitating natural selection.

### 4.1 Population‐level divergence

Across all candidate loci three Gunnison sage‐grouse populations generally clustered together (Gunnison Basin, Crawford, and Cimarron) and three of the populations stand out as holding signatures of potential divergent selection (San Miguel, Piñon Mesa, and Dove Creek; Figure 4c). It is interesting that the three populations with the most similar habitat conditions and in closest proximity are those that cluster at putatively divergent loci. In general, the shrub composition at Gunnison Basin, Cimarron, and Crawford is dominated by big sagebrush cover with patches of oakbrush and juniper (Gunnison sage‐grouse Rangewide Steering Committee, 2005). San Miguel and Dove Creek are both characterized by patchy big sagebrush habitat, fragmented by agriculture in Dove Creek, whereas San Miguel lowlands are dominated by low sagebrush cover. The shrub composition at Piñon Mesa varies along an elevation gradient; from low elevations dominated by sagebrush cover, saltbush, and greasewood; to piñon–juniper woodlands at mid elevations, and oakbrush with patchy sagebrush cover and snowberry at higher elevations. The majority of candidate loci were identified in environmental association analyses so the apparent clustering by differences in environment is not surprising, though it does suggest support for adaptation to local environmental conditions. In particular, the signal of diversifying selection is strongest in the Dove Creek, San Miguel, and Piñon Mesa populations (Figure 4c). Previous population genetic studies show Crawford, Cimarron, and Gunnison Basin are the most genetically similar of the six populations (Oyler‐McCance et al., 2005). When we look at putatively neutral loci (Figure 4b), these three populations remain distinct from each other. The pattern illustrated with all candidate loci we present in this manuscript where these populations cluster together, may be reflective of adaptive similarity (Figure 4c).

### 4.2 Ecological importance of identified signals of selection

One of the top enrichment clusters included terms indicating detoxification (oxidoreductase activity) as a biological process potentially underlying adaptive divergence and identified the same four cytochrome P450 family genes in many of the gene sets (Table 4). Our findings are consistent with the previously identified signals of divergence in this gene family in sage‐grouse populations (Oh et al., 2019). Different species of sagebrush have different compositions and quantities of PSM (Frye, Connelly, Musil, & Forbey, 2013; Kelsey et al., 1982) and divergence at genes involved in PSM metabolism may reflect local adaptation to consuming different species or subspecies of sagebrush. Sage‐grouse are dietary specialists on sagebrush (Patterson, 1952). Because sage‐grouse have mechanisms to mitigate inhibitory action of PSM on digestive enzymes (Kohl, Connelly, Dearing, & Forbey, 2016), these genes could potentially be responsible for proteins or enzymes that aid in sagebrush digestion. The candidate SNPs associated with all cytochrome P450 gene regions were identified with one or more of the environmental association analyses: CYP4V2 with green‐up rate, CYP2R1 with the dryness index and fall precipitation, CYP4B1 with fall precipitation, and CYP2C23B with CTI, respectively (Table 3).

The fourth enrichment cluster indentified by DAVID (Table 4) contained gene sets dominated by members of the DEAD box helicase gene family, generally known to function within multiprotein cellular complexes to perform various processes involving RNA metabolism (Linder & Jankowsky, 2011). Of particular interest to our findings are the members of this gene family known to play a role in detecting viral RNA in the cytoplasm of chicken (Schoggins et al., 2011; Zhang et al., 2016). SETX (Table S10), a gene associated with candidate adaptive loci, has been implicated in response to viral pathogens as well, including West Nile virus in chicken (WNV; Miller et al., 2015). Signals of diversifying selection associated with putative genes involved in antiviral activity could indicate the populations may have had different exposure histories which may result in differing abilities to respond to viral pathogens. Though it has yet to affect Gunnison sage‐grouse specifically, WNV has impacted susceptible greater sage‐grouse populations (Naugle et al., 2004) and the virus has been reported in other species within the Gunnison sage‐grouse range at varying levels (see Table S13 for information on reported WNV incidence in populations), suggesting a potential for exposure.

The reduced representation approach used here allowed us to break the entire genome down into smaller pieces and obtain higher confidence genotypes for more individuals than we would have been able to obtain with whole‐genome resequencing. However, this resulted in a low density of SNPs (~16 SNPs/Mb), and many regions of the genome were not sampled (Tiffin & Ross‐Ibarra, 2014). Additionally, our use of a threshold for linkage between SNPs and gene regions (5 kbp) was much lower than LD blocks (~350 kbp) that contain multiple gene regions. Consequently, candidate SNPs are likely linked to more than one gene region, any of which could be the target of selection. Therefore, it is likely there are more regions of the genome under adaptive divergence and more processes involved.

### 4.3 Conservation implications

We have identified signals of adaptive divergence associated with potentially ecologically important genes and groups of genes which may underlie adaptive divergence among populations of Gunnison sage‐grouse. Populations with different functional genetic variants could potentially impact management and conservation decisions (Savolainen, Lascoux, & Merilä, 2013). Theoretically, gene flow can have either a positive or negative impact on local adaptation of populations (Slatkin, 1987; Wright, 1931). If populations are locally adapted, increasing gene flow could risk outbreeding depression (Edmands, 2007), especially if populations are small. This has been exemplified in populations of streamside salamanders with and without predators where gene flow constrained the evolution of effective antipredator behaviors (Storfer & Sih, 1998). On the other hand, there have been many documented examples of gene flow promoting natural selection by increasing local genetic variation (Frankham, 2015; Miller, Poissant, Hogg, & Coltman, 2012), the new genetic variants may allow the population to respond to the local environmental conditions and potentially occupy new niche space (Aitken & Whitlock, 2013; Lenormand, 2002). However, there is still much to understand about the relative contributions of gene flow and natural selection to local adaptation (Kawecki & Ebert, 2004). The potential adaptive divergence associated with local adaptation to different sagebrush species (cytochrome P450 gene family) and response to viral pathogens (some DEAD box helicase family members and SETX) observed in Gunnison sage‐grouse suggest that individuals from one population may be less fit in the environment of a differently adapted population. Alternatively, movement of different genetic variants underlying these potentially important traits could facilitate local adaptation to viral pathogen response or PSM digestion in the distinct populations. Given that translocation has been one of the conservation strategies employed for the species (United States Fish & Wildlife Service, 2014), these findings could guide selection of appropriate source and recipient populations if future translocation efforts were to occur. While the samples used for this study were all collected prior to any translocation efforts, additional investigations (with more recently collected samples) are needed to evaluate whether putative adaptive allelic variants have been inadvertently diluted.

Similarly, if different populations are adapted to different species of sagebrush, habitat restoration efforts may require location specific sagebrush species as a seed source. Guidelines on seed and plant transfer zones for sagebrush species and subspecies have been based on moisture and elevation gradients in the past (Mahalovich & McArthur, 2004), which may result in planting a species or subspecies to which the local population is maladapted. Although matching the local sagebrush type during restoration could be important, efforts to do so could be complicated because seed sources for different sagebrush species or subspecies are not always available and factors involved in establishment of seedlings are just starting to be understood (Brabec, Germino, & Richardson, 2016).

Captive‐rearing of sage‐grouse has been attempted in recent years (Apa & Wiechman, 2015). Knowledge of adaptive differences could guide selection of targeted populations for release of captive‐reared birds. In the case of sagebrush digestion or disease response, releasing individuals with maladapted genotypes could not only result in wasted effort and resources, but may even lead to further reduction of average population fitness.

In conserving species with fragmented ranges and declining populations, restoration of gene flow between isolated groups is a common objective. Our findings suggest increasing gene flow between Gunnison sage‐grouse may require careful consideration of local adaptation. On the other hand, locally adapted variation might persist in the face of gene flow (Fitzpatrick, Gerberich, Kronenberger, Angeloni, & Funk, 2015) and the existence of adaptive environmental clines suggests gene flow via assisted migration can facilitate adaptive responses to climate change (Kelly & Phillips, 2016). We would be remiss to not acknowledge the potential for false positives in our analyses, however. Our outlier analysis methods generally control for demographic structure (i.e., incorporation of a kinship matrix or nonparametric approaches), though observed differentiation could still be a result of background selection, or linkage of neutrally evolving sites to sites under purifying selection (Shafer et al., 2015).

## 5 CONCLUSION

Our results are consistent with the hypothesis of adaptive divergence among populations of Gunnison sage‐grouse for potentially ecologically important metabolic phenotypes. This study takes the first step in understanding and characterizing local adaptation within populations of Gunnison sage‐grouse. The correlative approach we used assumes high‐frequency alleles in a population correspond to a higher fitness phenotype locally. This relationship could be confirmed or further probed through genomic methods that more directly evaluate fitness effects and function (Carneiro et al., 2014; Prasad et al., 2013). We used historical samples and publicly available geospatial data sets. More insight from our historical samples could be obtained by using the gene families as the subject of resequencing, or target enrichment, to identify functional variants supporting a putative role in adaptation and confirming signals of selection on a larger sample size (Jones & Good, 2016). Many approaches used to draw more direct lines between the underlying genetic controls and phenotype, such as quantitative trait analysis (Kearsey, 1998), and gene expression and/or reciprocal transplant studies (Kawecki & Ebert, 2004), may be attractive options to provide a phenotype link, especially given that many loci of varying effect size underlie adaptive divergence (Rockman, 2012). However, these strategies are unlikely feasible due to difficulty in generating large segregating populations in captivity and given federal protection of the species under the Endangered Species Act. Genome‐wide association studies (GWAS), on the other hand, can also identify genetic regions underlying phenotypes and can be accomplished without the use of captive populations making it a much more likely approach for future studies investigating local adaptation in Gunnison sage‐grouse. Nevertheless, our results have provided many avenues for future investigations of adaptation for this avian species of conservation concern.

## CONFLICT OF INTEREST

The authors have no conflicts of interest to declare.

## AUTHORS’ CONTRIBUTION

S.J.Z, S.J.O.M., C.L.A., and K.P.O. designed research. S.J.Z. performed research. R.S.C. performed bioinformatics. K.P.O. contributed new reagents or analytical tools. S.J.Z wrote the manuscript and analyzed the data. All authors contributed to manuscript editing.

## Data Availability

Genomic sequencing data for this study have been deposited in GenBank (biosample accession numbers: SAMN1084489–SAMN10844548; bioproject number: PRJNA517770). SNP genotypes for this study were deposited in the U.S. Geological Survey ScienceBasehttps://doi.org/10.5066/592. (Zimmerman, Aldridge, Oh, Cornman, & Oyler‐McCance, 2019)
