# Supplementary material for: Signatures of adaptive divergence among populations of an avian species of conservation concern
Source: Evol Appl. 2019 Jul 9;12(8):1661–77. doi: 10.1111/eva.12825 (PMC6708427; doi:10.1111/eva.12825)
Supplement: Supplementary file 1 [file EVA-12-1661-s001.docx]

**Supporting Information for:**

**Signatures of adaptive divergence among populations of an avian species with small effective population size at a small geographic scale**

**Table of Contents:**

| Appendix S1. Covariates and spatial data  processing | Page 2 |
| --- | --- |
| Appendix S2. Relatedness of available samples and  STRUCTURE plots. | Page 7 |
| Appendix S3. Scree and axis loading plots for the  Pacadapt analysis. | Page 8 |
| Appendix S4. Comparison of assumed population  differentiation patterns between SNPs and  microsatellites. | Page 9 |
| Appendix S5. Partial RDA scree plot | Page 10 |
| Appendix S6. Partial RDA biplot | Page 11 |
| Appendix S7. Overlap of candidate loci from  multiple analyses. | Page 12 |
| Appendix S8. Linkage disequilibrium decay. | Page 13 |
| Appendix S9. Full gene names for acronyms. | Page 14 |
| Appendix S10. Locus specific summary of  candidate SNPs and effects on putative  adaptive genes. | Page 22 |
| Appendix S12. Plots produced from individual  PCA analysis with each covariate evaluated. | Page 35 |
| Appendix S13. West Nile virus detection in  counties with Gunnison sage-grouse populations. | Page 36 |

Appendix S1. Covariates and Spatial Data Processing

We calculated a dryness index (DRI) and growing degree days (GDD) from Daymet (Thornton et al. 2017) data (see below for calculations). Individual tiles covering the study area (tiles 11376, 11377, 11556, 11557, 11558) were obtained for the time period 1997 to 2005 in the form of a NETcdf file. NETcdf files were converted to raster format and joined into a single surface (Python scripts available at <https://daymet.ornl.gov/tools.html>). GDD was calculated in ArcGIS10.1 by finding the average temperature, and counting the number of days in each year between 1 March and 31 August with temperatures greater than 5°C. The final GDD raster layer is an average across 9 years (1977 – 2005) to represent contemporary conditions. DRI was calculated by dividing GDD by the cumulative precipitation between 1 March and 31 August for each year. The final DRI raster layer is an average of 9 years. Our habitat variables were obtained from 30-m resolution Landfire data and processed in ArcGIS 10.1. We broke the cover types up into all sagebrush cover, low sagebrush cover, big sagebrush cover, and conifer cover, reclassifying a binary raster for each variable with the target variable given a 1 and everything else a 0. At the original 30-m resolution, cover type is presence/absence based. We were also interested in the configuration of conifer cover. To assess configuration we first had to convert the 30-m presence/absence conifer cover raster to point data. Next we applied a nearest neighbor analysis using the “near” tool in the analysis tools of arcMap. The output was then converted back to a raster and the nearest neighbor index was calculated by dividing the raster output by the mean distance (0.5*sqrt(total area/# of points in the distribution). Values less than 1 are considered clustered, while values greater than one are considered dispersed. The phenology tool (Talbert et al., 2013) was used to vegetation index values at different points on the phenology curve from MODIS normalized difference vegetation index (NDVI) data: green-up (beginning of growing season), brown-down (end of growing season), green-up rate (left derivative of the phenology curve), brown-down rate (right derivative of the phenology curve), and season length. Values for the years 2000 to 2010 were averaged for season 1 (the onset of growth). All spatial analyses were performed in ArcGIS10.1 unless stated otherwise. We obtained 30-year normals for annual and monthly rainfall, maximum temperature, minimum temperature, mean temperature, average dewpoint (the temperature below which water droplets begin to form; giving a sense of the amount of moisture in the air), minimum and maximum vapor pressure (the atmospheric pressure which is exerted by water vapor; a measure of humidity) at a resolution of 800 m from PRISM Climate Group (<http://prism.oregonstate.edu>). The monthly estimates were averaged into spring (average of April, May, and June), summer (average of July, August, September), fall (average of October, November, December) and winter (average of January, February, March) seasons according to the Farmer’s Almanac (https://www.farmersalmanac.com/the-seasons). We also averaged across nesting/leking (March, April, May), brood rearing (June, July, August, September), and winter (October, November, December, January, February, March) seasons of habitat use. Values were extracted from rasters with a 1-km radius for each lek. Values for leks were averaged across populations.

Table S1.1. Covariates included in tests for outlier loci.

| Covariate | Spatial Source | Derived |
| --- | --- | --- |
| Elevation | DEM |  |
| Dominant Shrub | Listing Decision |  |
| Brown-Down | phenology tool | NDVI from MODIS |
| Brown-Down Rate | phenology tool | NDVI from MODIS |
| Green-Up | phenology tool | NDVI from MODIS |
| Green-Up Rate | phenology tool | NDVI from MODIS |
| Season Length | phenology tool | NDVI from MODIS |
| Low Sage | Landfire |  |
| Conifer | Landfire |  |
| Big Sage | Landfire |  |
| All Sage | Landfire |  |
| Growing Degree Days | Daymet |  |
| Dryness Index | Daymet |  |
| Conifer Configuration | Landfire | nearest neighbor index |
| Precipitation |  |  |
| Annual | PRISM |  |
| Spring | PRISM | ave. of Apr, May, Jun |
| Summer | PRISM | ave. of Jul, Aug, Sep |
| Fall | PRISM | ave. of Oct, Nov, Dec |
| Winter | PRISM | ave. of Jan, Feb, Mar |
| Life Stage Precipitation | |  |
| Nesting | PRISM | ave. Mar, Apr, May |
| Brood Rearing | PRISM | ave. Jun, Jul, Aug, Sep |
| Winter | PRISM | ave. Oct, Nov, Dec, Jan, Feb |
| Maximum Temperature | |  |
| Annual | PRISM |  |
| Spring | PRISM | ave. of Apr, May, Jun |
| Summer | PRISM | ave. of Jul, Aug, Sep |
| Fall | PRISM | ave. of Oct, Nov, Dec |
| Winter | PRISM | ave. of Jan, Feb, Mar |
| Life Stage Maximum Temperature | |  |
| Nesting | PRISM | ave. Mar, Apr, May |
| Brood Rearing | PRISM | ave. Jun, Jul, Aug, Sep |
| Winter | PRISM | ave. Oct, Nov, Dec, Jan, Feb |
| Dew Point |  |  |
| Annual | PRISM |  |
| Spring | PRISM | ave. of Apr, May, Jun |
| Summer | PRISM | ave. of Jul, Aug, Sep |
| Fall | PRISM | ave. of Oct, Nov, Dec |
| Winter | PRISM | ave. of Jan, Feb, Mar |
| Life Stage Dew Point | |  |
| Nesting | PRISM | ave. Mar, Apr, May |
| Brood Rearing | PRISM | ave. Jun, Jul, Aug, Sep |
| Winter | PRISM | ave. Oct, Nov, Dec, Jan, Feb |
| Mean Temperature | |  |
| Annual | PRISM |  |
| Spring | PRISM | ave. of Apr, May, Jun |
| Summer | PRISM | ave. of Jul, Aug, Sep |
| Fall | PRISM | ave. of Oct, Nov, Dec |
| Winter | PRISM | ave. of Jan, Feb, Mar |
| Life Stage Mean Temperature | |  |
| Nesting | PRISM | ave. Mar, Apr, May |
| Brood Rearing | PRISM | ave. Jun, Jul, Aug, Sep |
| Winter | PRISM | ave. Oct, Nov, Dec, Jan, Feb |
| Minimum Temperature | |  |
| Annual | PRISM |  |
| Spring | PRISM | ave. of Apr, May, Jun |
| Summer | PRISM | ave. of Jul, Aug, Sep |
| Fall | PRISM | ave. of Oct, Nov, Dec |
| Winter | PRISM | ave. of Jan, Feb, Mar |
| Life Stage Minimum Temperature | |  |
| Nesting | PRISM | ave. Mar, Apr, May |
| Brood Rearing | PRISM | ave. Jun, Jul, Aug, Sep |
| Winter | PRISM | ave. Oct, Nov, Dec, Jan, Feb |
| Maximum Vapor Pressure | |  |
| Annual | PRISM |  |
| Spring | PRISM | ave. of Apr, May, Jun |
| Summer | PRISM | ave. of Jul, Aug, Sep |
| Fall | PRISM | ave. of Oct, Nov, Dec |
| Winter | PRISM | ave. of Jan, Feb, Mar |
| Life Stage Maximum Vapor Pressure | |  |
| Nesting | PRISM | ave. Mar, Apr, May |
| Brood Rearing | PRISM | ave. Jun, Jul, Aug, Sep |
| Winter | PRISM | ave. Oct, Nov, Dec, Jan, Feb |
| Minimum Vapor Pressure | |  |
| Annual | PRISM |  |
| Spring | PRISM | ave. of Apr, May, Jun |
| Summer | PRISM | ave. of Jul, Aug, Sep |
| Fall | PRISM | ave. of Oct, Nov, Dec |
| Winter | PRISM | ave. of Jan, Feb, Mar |
| Life Stage Minimum Vapor Pressure | |  |
| Nesting | PRISM | ave. Mar, Apr, May |
| Brood Rearing | PRISM | ave. Jun, Jul, Aug, Sep |
| Winter | PRISM | ave. Oct, Nov, Dec, Jan, Feb |

Table S1.2. Correlation coefficients for uncorrelated variables included as covariates in tests for outlier loci.

|  | spring_ppt | fall_ppt | spring_tmax | winter_vpdmax | cti | gur | bs | dri |
| --- | --- | --- | --- | --- | --- | --- | --- | --- |
| spring_ppt | 1.00 |  |  |  |  |  |  |  |
| fall_ppt | 0.52 | 1.00 |  |  |  |  |  |  |
| spring_tmax | -0.35 | 0.23 | 1.00 |  |  |  |  |  |
| winter_vpdmax | 0.68 | 0.63 | 0.41 | 1.00 |  |  |  |  |
| cti | -0.39 | 0.19 | 0.61 | 0.23 | 1.00 |  |  |  |
| gur | 0.31 | 0.47 | -0.64 | -0.23 | -0.29 | 1.00 |  |  |
| bs | 0.09 | 0.11 | 0.69 | 0.56 | -0.01 | -0.65 | 1.00 |  |
| dri | -0.62 | -0.28 | 0.39 | -0.47 | -0.09 | -0.30 | 0.14 | 1.00 |

Table S1.3. Loadings of variables correlated < |0.7| onto principal components.

| Covariate | PC1 | PC2 | PC3 |
| --- | --- | --- | --- |
| spring_ppt | -0.13 | 0.53 | -0.12 |
| fall_ppt | -0.03 | 0.34 | 0.29 |
| spring_tmax | 0.53 | -0.05 | 0.25 |
| winter_vpdmax | 0.28 | 0.54 | 0.15 |
| cti | 0.19 | -0.07 | 0.76 |
| gur | -0.53 | 0.15 | 0.11 |
| bs | 0.54 | 0.21 | -0.48 |
| dri | 0.13 | -0.50 | -0.06 |

Figure S1.1. Covariate maps of the eight uncorrelated covariates used in the association analyses: a = spring precipitation (cm), b = fall precipitation (cm), c = spring maximum temperature (°C), d = winter maximum vapor pressure deficit (hPa), e = compound topographic index, f = green-up rate, g = big sagebrush cover (proportion of pixels with sagebrush cover), h = dryness index (# days with average temperature > 5 °C in growing season per cm of total spring precipitation). Pixel resolution = 1 km^2^.


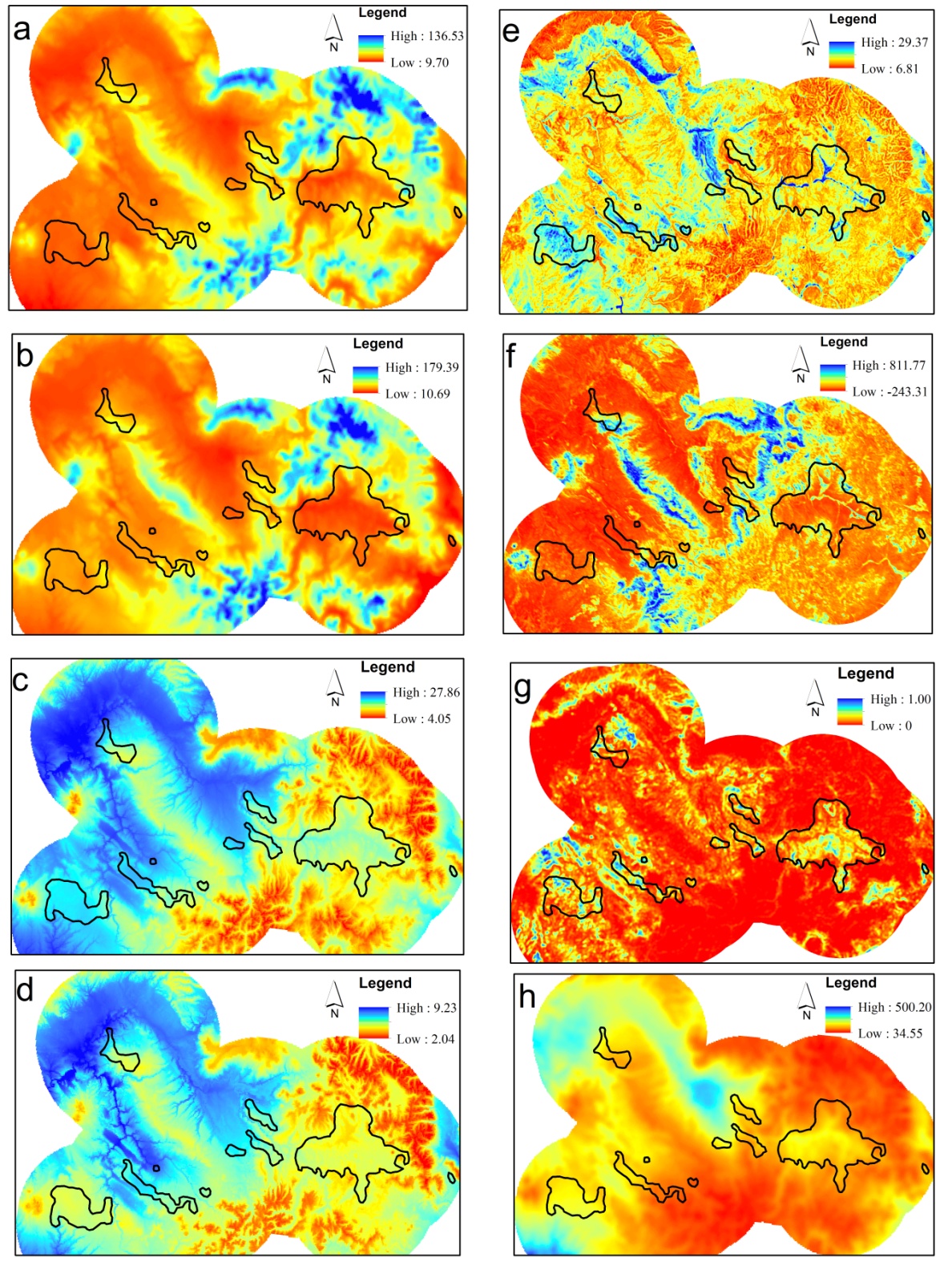


Appendix S2 Relatedness and STRUCTURE analysis

Table S2.1. Mean, minimum, and maximum pairwise relatedness scores according to Lynch and Ritland (1999) for the samples selected for genomic library preparation (“Selected”) and all available samples (“All Possible”).

| Statistic | Selected | All Possible |
| --- | --- | --- |
| Mean | -0.009 | -0.002 |
| Minimum | -0.138 | -0.198 |
| Maximum | 0.354 | 0.468 |

**
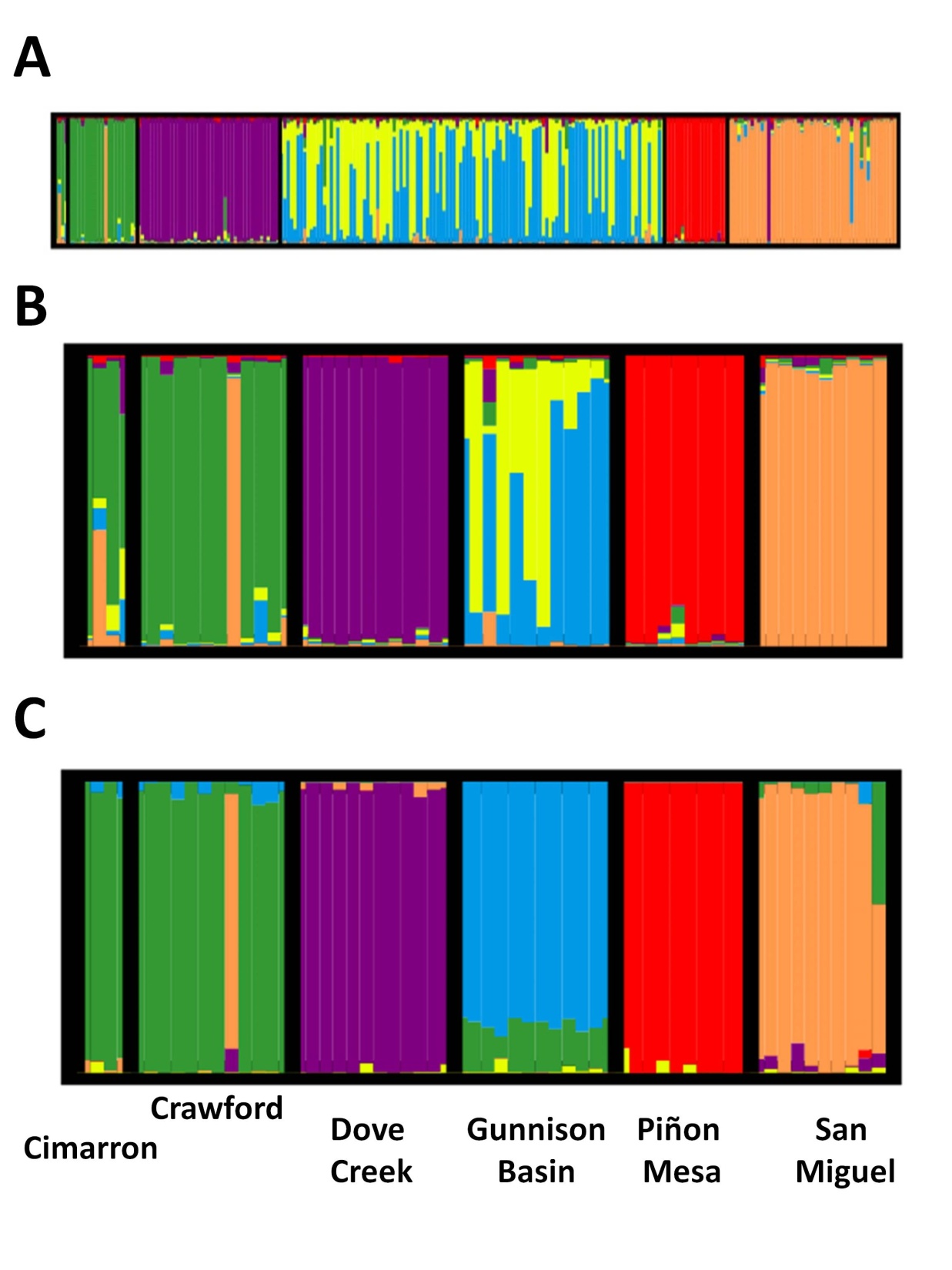
**

Figure S2.2. Comparison optimal number of populations (K = 6; Evanno et al. 2005) based on a Bayesian clustering analysis, STRUCTURE (Pritchard et al. 2000) using 22 microsatellite loci, all 264 individual samples, an admixture model, a burn-in of 250,000 and 300,000 Markov chain Monte Carlo (MCMC) iterations testing 20 replicates of each hypothetical number of populations from K=1 to K=10 (A), the samples that were selected for library preparation (B), the barplot from a STRUCTURE analysis as described above for K = 6 using the SNP data for the selected individuals (C).


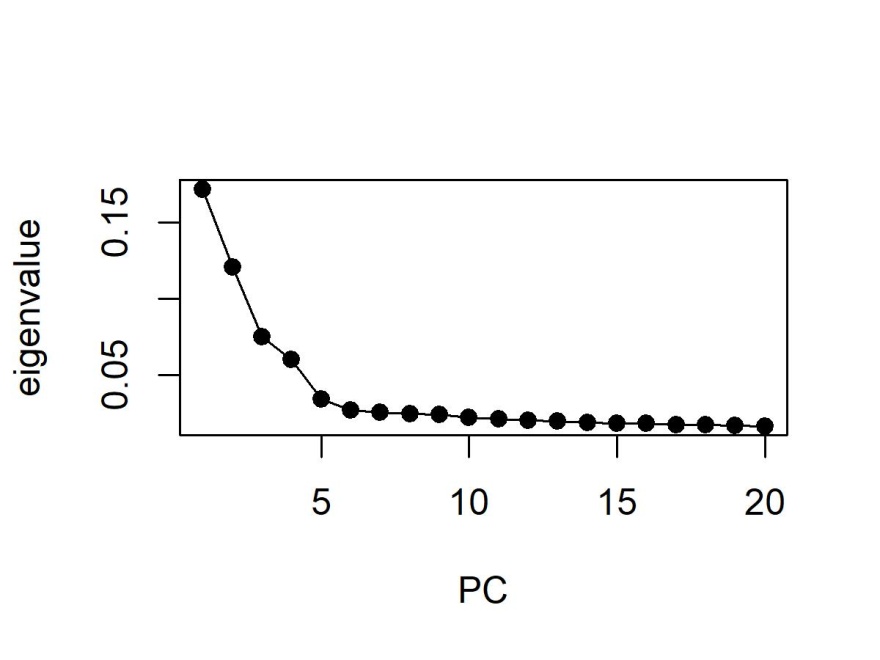


Figure S3.1. Scree plot for pcadapt. Proportion of variance (eigenvalue) accounted for by the first 20 PCs. The first 5 were retained for analysis.


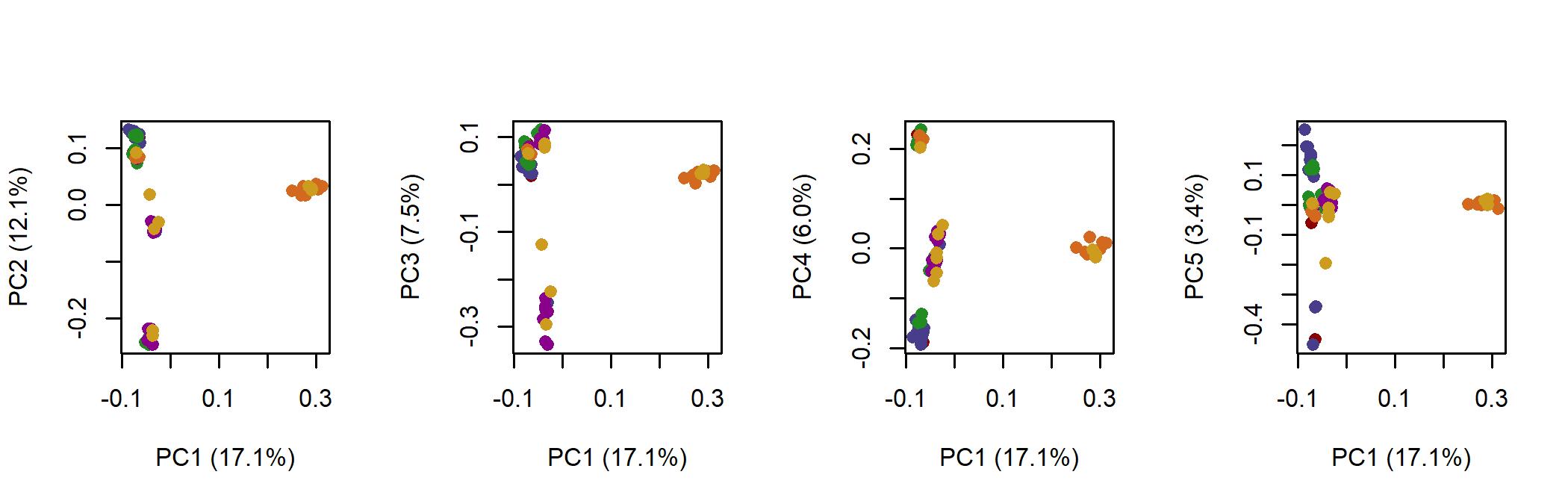


Figure S3.2. Plots of the first 5 principal components (PC) from pcadapt. Each point is an individual color coded by the population in which it was sampled. Cimarron = red, Crawford = blue, Dove Creek = green, Gunnison Basin = purple, Piñon Mesa = orange, San Miguel = yellow.
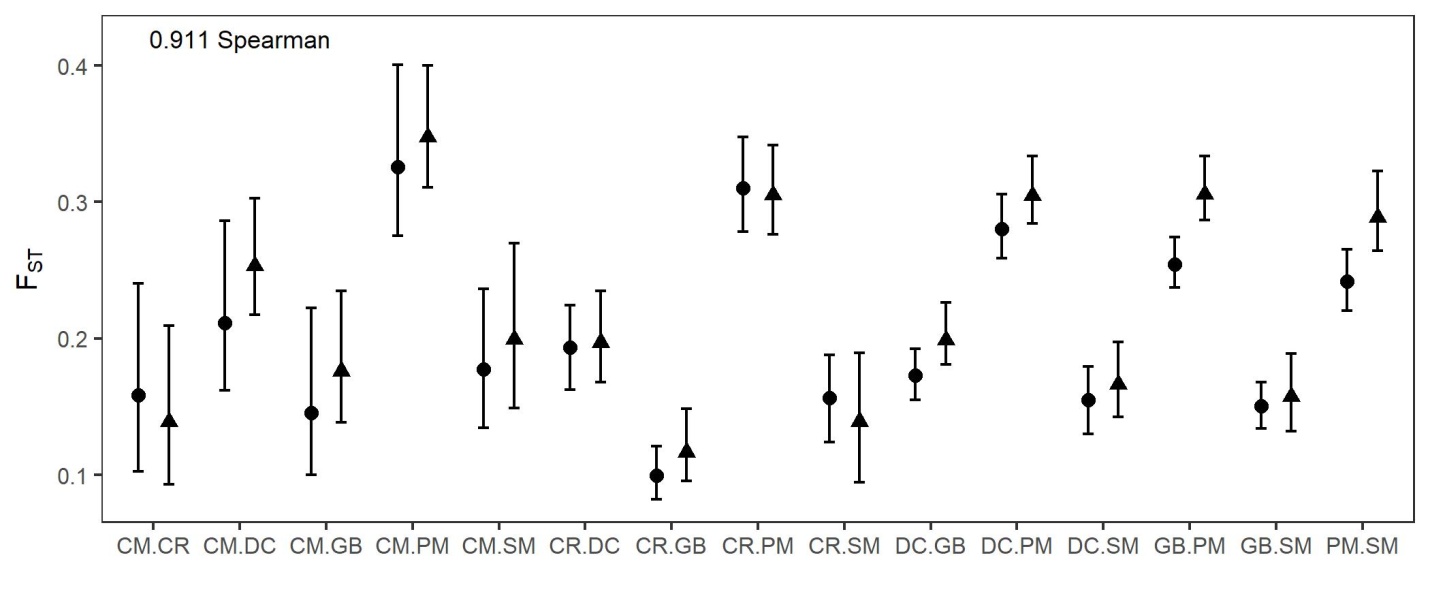


Appendix Figure S4. Comparison of F_ST_ values with confidence intervals from microsatellite and SNP loci. Values were estimated as in Weir and Cockerham (1984) for 254 Gunnison sage-grouse individuals and 22 microsatellites (●) and 60 individuals (a subset of the 254) with 4,933 SNP loci (▲). Populations in pair-wise comparisons are abbreviated along the x-axis: CM = Cimarron, CR = Crawford, DC = Dove Creek, GB = Gunnison Basin, PM = Piñon Mesa, SM = San Miguel; CM.CR = F_ST_ between Cimarron and Crawford. Spearman rank and Pearson correlation coefficient of F_ST_ from the two marker sets is included in the upper left-hand corner.


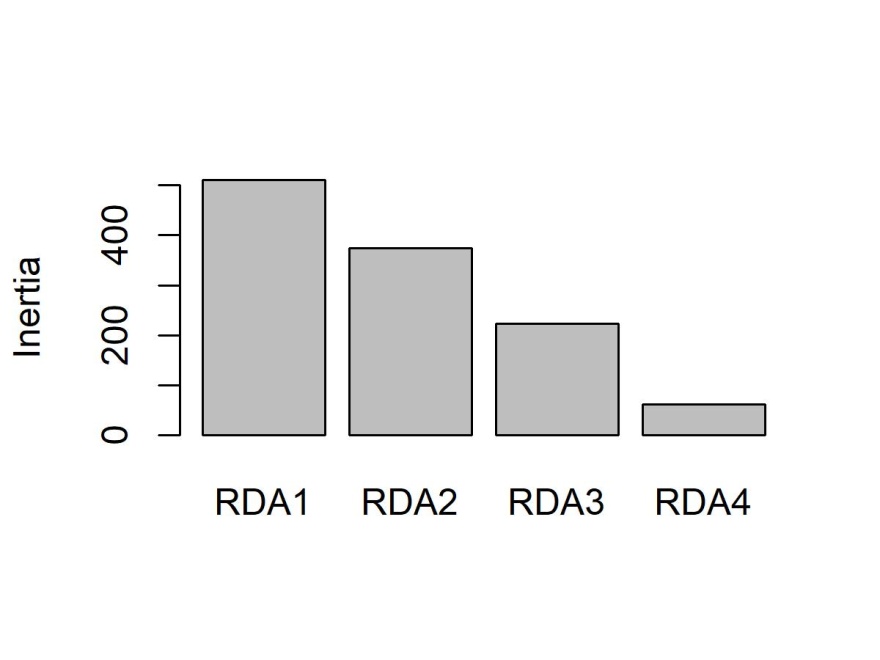


Figure S5. Partial RDA scree plot. Amount of variance (inertia) accounted for by each of the 4 RDA axes.


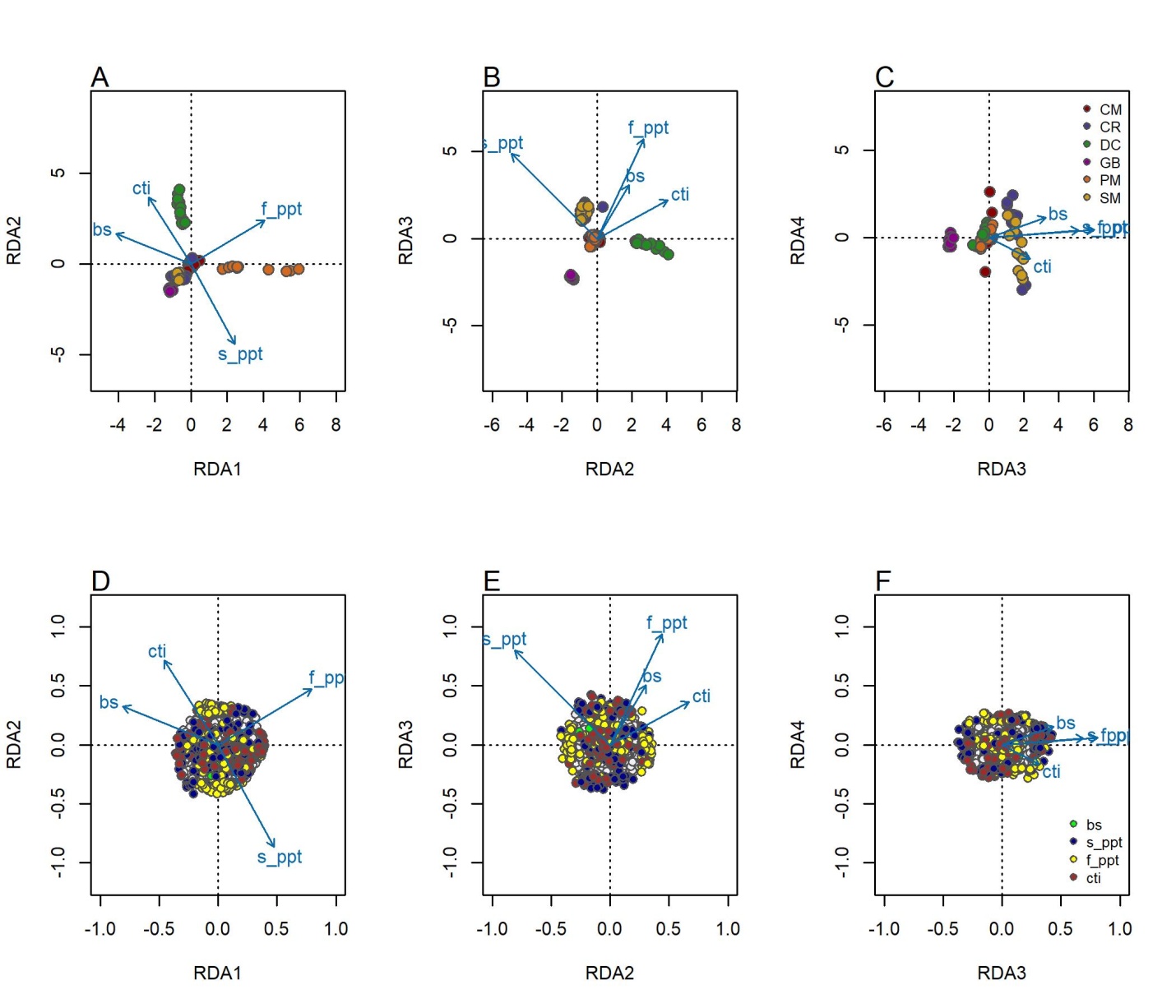


Figure S6. Genome-wide multivariate genome-environmental associations in a partial redundancy analysis (RDA). In panels A – C, populations are abbreviated in the legend: CM = Cimarron, CR = Crawford, DC = Dove Creek, GB = Gunnison Basin, PM = Piñon Mesa, SM = San Miguel. Vector labels are abbreviated as follows: bs = proportion of big sagebrush, cti = compound topographic index, f_ppt = fall precipitation, s_ppt = spring precipitation.

Appendix Table S7. Overlap of candidate loci from multiple analyses

Counts of SNPs in common identified in pairs of outlier and environmental association analyses.

|  | pcadapt | xtx | rda | bs | cti | dri | fallppt | gur | springppt | springtmax | wintervpdmax | pc1 | pc2 | pc3 |
| --- | --- | --- | --- | --- | --- | --- | --- | --- | --- | --- | --- | --- | --- | --- |
| pcadapt | 156 |  |  |  |  |  |  |  |  |  |  |  |  |  |
| xtx | 29 | 76 |  |  |  |  |  |  |  |  |  |  |  |  |
| rda | 60 | 34 | 602 |  |  |  |  |  |  |  |  |  |  |  |
| bs | 4 | 3 | 5 | 36 |  |  |  |  |  |  |  |  |  |  |
| cti | 1 | 4 | 1 | 0 | 27 |  |  |  |  |  |  |  |  |  |
| dri | 5 | 12 | 10 | 0 | 0 | 45 |  |  |  |  |  |  |  |  |
| fallppt | 2 | 4 | 3 | 0 | 0 | 0 | 26 |  |  |  |  |  |  |  |
| gur | 2 | 0 | 0 | 0 | 0 | 0 | 0 | 30 |  |  |  |  |  |  |
| springppt | 1 | 2 | 3 | 0 | 0 | 2 | 0 | 0 | 34 |  |  |  |  |  |
| springtmax | 1 | 0 | 2 | 0 | 0 | 0 | 0 | 0 | 0 | 14 |  |  |  |  |
| wintervpdmax | 4 | 5 | 3 | 0 | 0 | 0 | 13 | 0 | 1 | 0 | 28 |  |  |  |
| pc1 | 6 | 2 | 10 | 2 | 0 | 0 | 0 | 0 | 0 | 8 | 0 | 40 |  |  |
| pc2 | 1 | 2 | 2 | 0 | 0 | 0 | 0 | 0 | 4 | 0 | 0 | 0 | 13 |  |
| pc3 | 2 | 6 | 3 | 0 | 11 | 0 | 0 | 0 | 0 | 0 | 1 | 0 | 0 | 43 |


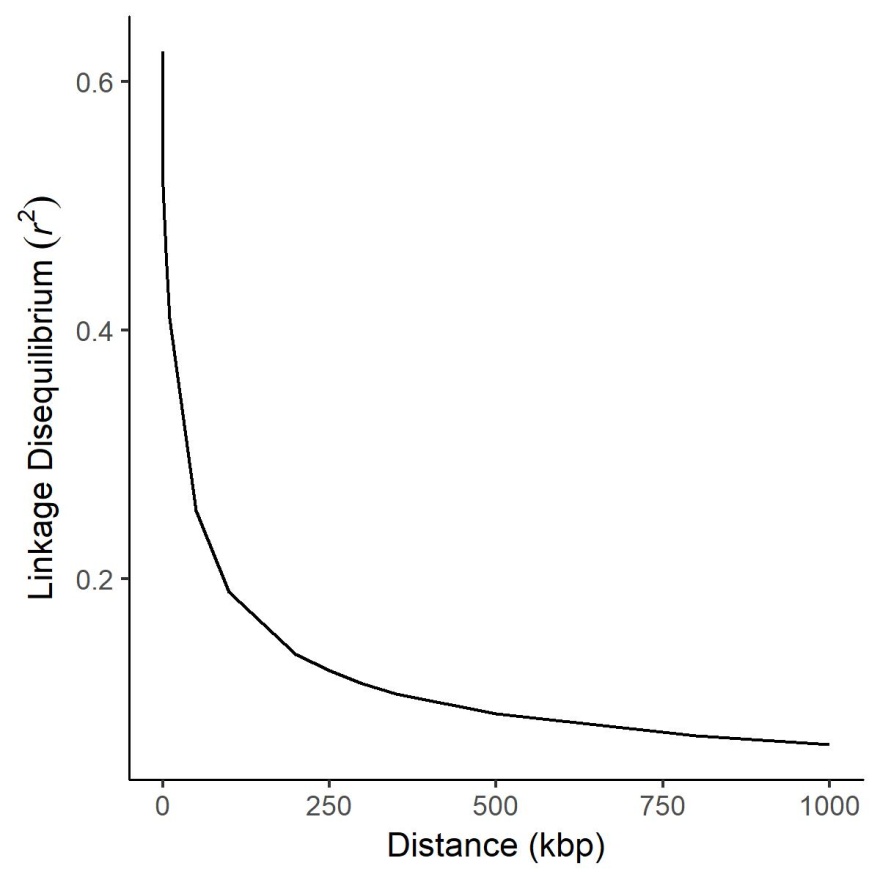


Appendix Figure S8. Linkage disequilibrium (LD), as measured by *r*^2^, for Gunnison sage-grouse after phasing SNPs using BEAGLE 5.0 (setting N_E_ to 1,000 to indicate our data are from a small and inbred population as recommended by Browning & Browning (2007)). We calculated LD in vcftools (-hap-r2 command) at multiple distances, from SNPs 10 bp to 1Mbp apart. Distances over which *r*^2^ was calculated are indicated on the x-axis in kilobase pairs (kbp).

sAppendix Table S9. Gene codes and associated full gene names that candidate adaptive SNPs from outlier analyses are linked to for Gunnison sage-grouse populations.

| Code | Gene Name |
| --- | --- |
| ABCC9 | ATP-binding cassette, sub-family C (CFTR/MRP), member 9 |
| ABRACL | ABRA C-terminal like |
| ACAD11 | acyl-CoA dehydrogenase family, member 11 |
| ACOT4 | acyl-CoA thioesterase 4 |
| ACSF3 | acyl-CoA synthetase family member 3 |
| ACSL1 | acyl-CoA synthetase long-chain family member 1 |
| ACTR2 | ARP2 actin-related protein 2 homolog (yeast) |
| ADAMTS20 | ADAM metallopeptidase with thrombospondin type 1 motif, 20 |
| ADCY5 | adenylate cyclase 5 |
| AGO2 | argonaute RISC catalytic component 2 |
| AGPAT9 | 1-acylglycerol-3-phosphate O-acyltransferase 9 |
| ALMS1 | Alstrom syndrome 1 |
| ALOX5 | arachidonate 5-lipoxygenase |
| ANKRD10 | ankyrin repeat domain 10 |
| ANKRD13C | ankyrin repeat domain 13C |
| ANKRD27 | ankyrin repeat domain 27 |
| ANKRD44 | ankyrin repeat domain 44 |
| ANKRD50 | ankyrin repeat domain 50 |
| ANKS1B | ankyrin repeat and sterile alpha motif domain containing 1B |
| ANO4 | anoctamin 4 |
| APPL2 | adaptor protein, phosphotyrosine interaction, PH domain and leucine zipper containing 2 |
| ARL5B | ADP-ribosylation factor-like 5B |
| ARPP19 | cAMP-regulated phosphoprotein, 19kDa |
| ASMTL | acetylserotonin O-methyltransferase-like |
| ASXL3 | additional sex combs like 3, transcriptional regulator |
| ATXN2 | ataxin 2 |
| AURKA | aurora kinase A |
| AURKAIP1 | aurora kinase A interacting protein 1 |
| BACH2 | BTB domain and CNC homolog 2 |
| BCL11A | B-cell CLL/lymphoma 11A (zinc finger protein) |
| BCL11A | B-cell CLL/lymphoma 11A (zinc finger protein) |
| BEGAIN | brain enriched guanylate kinase associated |
| BEST3 | bestrophin 3 |
| BICD1 | bicaudal D homolog 1 (Drosophila) |
| BIRC6 | baculoviral IAP repeat containing 6 |
| BMP2K | BMP2 inducible kinase |
| BMPER | BMP binding endothelial regulator |
| BRD2 | bromodomain containing 2 |
| BRINP3 | bone morphogenetic protein/retinoic acid inducible neural-specific 3 |
| BRWD1 | bromodomain and WD repeat domain containing 1 |
| BUB1 | BUB1 mitotic checkpoint serine/threonine kinase |
| C10ORF112 | chromosome 2 open reading frame, human C10orf112 |
| C1H21ORF33 | chromosome 1 open reading frame, human C21orf33 |
| C1H3ORF38 | chromosome 1 open reading frame, human C3orf38 |
| C21H1ORF159 | chromosome 21 open reading frame, human C1orf159 |
| C4H4ORF27 | chromosome 4 open reading frame, human C4orf27 |
| C5 | complement component 5 |
| C5H11ORF58 | chromosome 5 open reading frame, human C11orf58 |
| CALCR | calcitonin receptor |
| CAPN6 | calpain 6 |
| CAPN7 | calpain 7 |
| CAPRIN1 | cell cycle associated protein 1 |
| CAT | catalase |
| CC2D1B | coiled-coil and C2 domain containing 1B |
| CCDC28A | coiled-coil domain containing 28A |
| CCDC28A | coiled-coil domain containing 28A |
| CCDC47 | coiled-coil domain containing 47 |
| CD82 | CD82 molecule |
| CD86 | CD86 molecule |
| CDCP1 | CUB domain containing protein 1 |
| CENPW | centromere protein W |
| CEP135 | centrosomal protein 135kDa |
| CERS3 | ceramide synthase 3 |
| CHAT | choline O-acetyltransferase |
| CHAT | choline O-acetyltransferase |
| CHKA | choline kinase alpha |
| CHPT1 | choline phosphotransferase 1 |
| CLCN3 | chloride channel, voltage-sensitive 3 |
| CLEC3B | C-type lectin domain family 3 member B |
| CMIP | c-Maf inducing protein |
| CMTR1 | cap methyltransferase 1 |
| CNTRL | centriolin |
| COL6A3 | collagen, type VI, alpha 3 |
| CORO1C | coronin, actin binding protein, 1C |
| COX10 | COX10 heme A:farnesyltransferase cytochrome c oxidase assembly factor |
| CRIM1 | cysteine rich transmembrane BMP regulator 1 (chordin-like) |
| CRYAA | crystallin, alpha A |
| CTH | cystathionine gamma-lyase |
| CYP2C23b | cytochrome P450, family 2, subfamily C, polypeptide 23b |
| CYP2R1 | cytochrome P450 family 2 subfamily R member 1 |
| CYP4B1 | cytochrome P450, family 4, subfamily B, polypeptide 1 |
| CYP4V2 | cytochrome P450 family 4 subfamily V member 2 |
| CYTH3 | cytohesin 3 |
| DAAM2 | dishevelled associated activator of morphogenesis 2 |
| DDX1 | DEAD (Asp-Glu-Ala-Asp) box helicase 1 |
| DDX10 | DEAD (Asp-Glu-Ala-Asp) box polypeptide 10 |
| DDX42 | DEAD (Asp-Glu-Ala-Asp) box helicase 42 |
| DENND5A | DENN/MADD domain containing 5A |
| DHRS3 | dehydrogenase/reductase (SDR family) member 3 |
| DNAJC13 | DnaJ heat shock protein family (Hsp40) member C13 |
| EAF2 | ELL associated factor 2 |
| EHHADH | enoyl-CoA, hydratase/3-hydroxyacyl CoA dehydrogenase |
| EIF1B | eukaryotic translation initiation factor 1B |
| EPAS1 | endothelial PAS domain protein 1 |
| EXD2 | exonuclease 3'-5' domain containing 2 |
| EXOC1 | exocyst complex component 1 |
| FADS2 | fatty acid desaturase 2 |
| FADS2 | fatty acid desaturase 2 |
| FAM105A | family with sequence similarity 105, member A |
| FAM126A | family with sequence similarity 126, member A |
| FAM126A | family with sequence similarity 126, member A |
| FAM210B | family with sequence similarity 210 member B |
| FGF13 | fibroblast growth factor 13 |
| FTL | ferritin, light polypeptide |
| FUCA2 | fucosidase, alpha-L- 2, plasma |
| GABRB3 | gamma-aminobutyric acid (GABA) A receptor, beta 3 |
| GARNL3 | GTPase activating Rap/RanGAP domain like 3 |
| GCOM1 | GRINL1A complex locus 1 |
| GIMD1 | GIMAP family P-loop NTPase domain containing 1 |
| GLRA3 | glycine receptor, alpha 3 |
| GNPTAB | N-acetylglucosamine-1-phosphate transferase, alpha and beta subunits |
| GPM6A | glycoprotein M6A |
| GRHL1 | grainyhead-like 1 (Drosophila) |
| GRIN2A | glutamate receptor, ionotropic, N-methyl D-aspartate 2A |
| GRM5 | glutamate receptor, metabotropic 5 |
| GTF2H1 | general transcription factor IIH subunit 1 |
| H2AFY | H2A histone family, member Y |
| HBP1 | HMG-box transcription factor 1 |
| HOOK1 | hook microtubule-tethering protein 1 |
| HPRT1 | hypoxanthine phosphoribosyltransferase 1 |
| HSD17B12 | hydroxysteroid (17-beta) dehydrogenase 12 |
| HSP90B1 | heat shock protein 90kDa beta (Grp94), member 1 |
| IL18RAP | interleukin 18 receptor accessory protein |
| IL1R2 | interleukin 1 receptor type 2 |
| INF2 | inverted formin, FH2 and WH2 domain containing |
| INPP5D | inositol polyphosphate-5-phosphatase, 145kDa |
| INPPL1 | inositol polyphosphate phosphatase-like 1 |
| IQCE | IQ motif containing E |
| IRAK4 | interleukin 1 receptor associated kinase 4 |
| IRF2 | interferon regulatory factor 2 |
| KCNMB4 | potassium calcium-activated channel subfamily M regulatory beta subunit 4 |
| KIAA1210 | KIAA1210 |
| KIAA1549L | KIAA1549-like |
| KIF13A | kinesin family member 13A |
| KIF18A | kinesin family member 18A |
| KIF23 | kinesin family member 23 |
| KRT20 | keratin 20 |
| LAMA5 | laminin, alpha 5 |
| LAMP3 | lysosomal associated membrane protein 3 |
| LGALSL | lectin, galactoside-binding-like |
| LOC100857837 | ethanolaminephosphotransferase 1-like |
| LOC101751732 | uncharacterized LOC101751732 |
| LOC419830 | uncharacterized LOC419830 |
| LOC422214 | nuclear cap binding protein subunit 2, 20kDa |
| LOC424998 | multiple EGF-like-domains 6-like |
| LOC427545 | histamine H3 receptor-like |
| LOC428693 | diacylglycerol O-acyltransferase 2-like |
| LOC768589 | baculoviral IAP repeat-containing protein 5.1-like |
| LOC771638 | putative short-chain dehydrogenase/reductase family 42E member 2-like |
| LRCH2 | leucine-rich repeats and calponin homology (CH) domain containing 2 |
| LRIT2 | leucine-rich repeat, immunoglobulin-like and transmembrane domains 2 |
| LRP2BP | LRP2 binding protein |
| LRP4 | low density lipoprotein receptor-related protein 4 |
| LYVE1 | lymphatic vessel endothelial hyaluronan receptor 1 |
| MAP2 | microtubule-associated protein 2 |
| MAP3K3 | mitogen-activated protein kinase kinase kinase 3 |
| MAP7D2 | MAP7 domain containing 2 |
| MARCH1 | membrane-associated ring finger (C3HC4) 1, E3 ubiquitin protein ligase |
| MARCH8 | membrane-associated ring finger (C3HC4) 8, E3 ubiquitin protein ligase |
| MAT1A | methionine adenosyltransferase I, alpha |
| MCM4 | minichromosome maintenance complex component 4 |
| MEP1A | meprin A subunit alpha |
| MICALL2 | MICAL like 2 |
| MMP16 | matrix metallopeptidase 16 (membrane-inserted) |
| MORF4L1 | mortality factor 4 like 1 |
| MSN | moesin |
| MSN | moesin |
| MTF1 | metal-regulatory transcription factor 1 |
| MYBPC3 | myosin binding protein C, cardiac |
| MYLIP | myosin regulatory light chain interacting protein |
| MYO1B | myosin IB |
| MYO1B | myosin IB |
| MYO1B | myosin IB |
| MYO1H | myosin IH |
| NAALAD2 | N-acetylated alpha-linked acidic dipeptidase 2 |
| NAB1 | NGFI-A binding protein 1 (EGR1 binding protein 1) |
| NADSYN1 | NAD synthetase 1 |
| NAP1L4 | nucleosome assembly protein 1-like 4 |
| NBAS | neuroblastoma amplified sequence |
| NCKAP1 | NCK-associated protein 1 |
| NDUFS3 | NADH:ubiquinone oxidoreductase core subunit S3 |
| NMBR | neuromedin B receptor |
| NSRP1 | nuclear speckle splicing regulatory protein 1 |
| NTPCR | nucleoside-triphosphatase, cancer-related |
| NUCKS1 | nuclear casein kinase and cyclin-dependent kinase substrate 1 |
| NUP205 | nucleoporin 205kDa |
| OPN4-1 | photopigment melanopsin-like |
| ORC1 | origin recognition complex subunit 1 |
| P2RY4 | pyrimidinergic receptor P2Y, G-protein coupled, 4 |
| PAK2 | p21 protein (Cdc42/Rac)-activated kinase 2 |
| PALLD | palladin, cytoskeletal associated protein |
| PARVA | parvin, alpha |
| PCDH12 | protocadherin 12 |
| PCDH9 | protocadherin 9 |
| PDE3B | phosphodiesterase 3B, cGMP-inhibited |
| PHACTR1 | phosphatase and actin regulator 1 |
| PHKA2 | phosphorylase kinase, alpha 2 (liver) |
| PHKA2 | phosphorylase kinase, alpha 2 (liver) |
| PI4K2B | Phosphatidylinositol 4-kinase type 2 beta; Phosphatidylinositol 4-kinase type 2-beta |
| PIK3CA | phosphatidylinositol-4,5-bisphosphate 3-kinase, catalytic subunit alpha |
| PKP2 | plakophilin 2 |
| PLCB1 | phospholipase C, beta 1 (phosphoinositide-specific) |
| PLEKHA7 | pleckstrin homology domain containing A7 |
| PLEKHG4 | pleckstrin homology domain containing, family G (with RhoGef domain) member 4 |
| PLIN3 | perilipin 3 |
| PLIN3 | perilipin 3 |
| POLR3B | polymerase (RNA) III (DNA directed) polypeptide B |
| PPARA | peroxisome proliferator-activated receptor alpha |
| PPP1R12A | protein phosphatase 1, regulatory subunit 12A |
| PRKAR1B | protein kinase, cAMP-dependent, regulatory, type I, beta |
| PRKDC | protein kinase, DNA-activated, catalytic polypeptide |
| PRRG1 | proline rich Gla (G-carboxyglutamic acid) 1 |
| PSMD1 | proteasome 26S subunit, non-ATPase 1 |
| PSMG4 | proteasome (prosome, macropain) assembly chaperone 4 |
| PTPLA | protein tyrosine phosphatase-like (proline instead of catalytic arginine), member A |
| PTPRT | protein tyrosine phosphatase, receptor type, T |
| PUM2 | pumilio RNA-binding family member 2 |
| PUS7L | pseudouridylate synthase 7 like |
| RAB11FIP4 | RAB11 family interacting protein 4 (class II) |
| RAP1GDS1L | rap1 GTP-GDP dissociation stimulator 1-like |
| RELN | reelin |
| RFC1 | replication factor C subunit 1 |
| RIC8B | RIC8 guanine nucleotide exchange factor B |
| RNF149 | ring finger protein 149 |
| RNF216 | ring finger protein 216 |
| RPIA | ribose 5-phosphate isomerase A |
| RPS6KC1 | ribosomal protein S6 kinase, 52kDa, polypeptide 1 |
| RPSA | ribosomal protein SA |
| RRP1B | ribosomal RNA processing 1 homolog B (S. cerevisiae) |
| RUFY3 | RUN and FYVE domain containing 3 |
| RWDD1 | RWD domain containing 1 |
| RWDD4 | RWD domain containing 4 |
| SCCPDH | saccharopine dehydrogenase (putative) |
| SDK1 | sidekick cell adhesion molecule 1 |
| SDR42E2 | short chain dehydrogenase/reductase family 42E, member 2 |
| SEC16A | SEC16 homolog A (S. cerevisiae) |
| SEC22A | SEC22 homolog A, vesicle trafficking protein |
| SEH1L | SEH1 like nucleoporin |
| SEPSECS | Sep (O-phosphoserine) tRNA:Sec (selenocysteine) tRNA synthase |
| SERAC1 | serine active site containing 1 |
| SETX | senataxin |
| SLC12A4 | solute carrier family 12 (potassium/chloride transporters), member 4 |
| SLC13A5 | solute carrier family 13 (sodium-dependent citrate transporter), member 5 |
| SLC25A22 | solute carrier family 25 (mitochondrial carrier: glutamate), member 22 |
| SLC2A8 | solute carrier family 2 (facilitated glucose transporter), member 8 |
| SLC39A11 | solute carrier family 39 member 11 |
| SLC39A12 | solute carrier family 39 (zinc transporter), member 12 |
| SLC9A4 | solute carrier family 9, subfamily A (NHE4, cation proton antiporter 4), member 4 |
| SMPD4 | sphingomyelin phosphodiesterase 4 |
| SMURF1 | SMAD specific E3 ubiquitin protein ligase 1 |
| SNAP47 | synaptosomal-associated protein, 47kDa |
| SPAG9 | sperm associated antigen 9 |
| SPATA17 | spermatogenesis associated 17 |
| SPRYD7 | SPRY domain containing 7 |
| SRD5A3 | steroid 5 alpha-reductase 3 |
| SRPX2 | sushi-repeat containing protein, X-linked 2 |
| SRRL | serine racemase-like |
| ST6GAL2 | ST6 beta-galactosamide alpha-2,6-sialyltranferase 2 |
| STAG1 | stromal antigen 1 |
| SYNE2 | spectrin repeat containing, nuclear envelope 2 |
| SYNE3 | spectrin repeat containing, nuclear envelope family member 3 |
| SYTL4 | synaptotagmin like 4 |
| TAB2 | TGF-beta activated kinase 1/MAP3K7 binding protein 2 |
| TBC1D19 | TBC1 domain family member 19 |
| TBC1D9 | TBC1 domain family, member 9 (with GRAM domain) |
| TBPL1 | TATA-box binding protein like 1 |
| TCEANC | transcription elongation factor A N-terminal and central domain containing |
| TCF21 | transcription factor 21 |
| TCF7 | transcription factor 7 (T-cell specific, HMG-box) |
| TFAP2D | transcription factor AP-2 delta (activating enhancer binding protein 2 delta) |
| TGFBRAP1 | transforming growth factor beta receptor associated protein 1 |
| TH | tyrosine hydroxylase |
| TJAP1 | tight junction associated protein 1 |
| TMA16 | translation machinery associated 16 homolog |
| TMEM242 | transmembrane protein 242 |
| TMEM59 | transmembrane protein 59 |
| TMEM68 | transmembrane protein 68 |
| TNFSF13B | tumor necrosis factor (ligand) superfamily, member 13b |
| TNRC18 | trinucleotide repeat containing 18 |
| TPCN3 | two-pore calcium channel 3 |
| TPP2 | tripeptidyl peptidase II |
| TRAPPC10 | trafficking protein particle complex 10 |
| TRAPPC11 | trafficking protein particle complex 11 |
| TRPM8 | transient receptor potential cation channel, subfamily M, member 8 |
| TRPM8 | transient receptor potential cation channel, subfamily M, member 8 |
| TSC1 | tuberous sclerosis 1 |
| TSNAXIP1 | translin-associated factor X interacting protein 1 |
| TSPAN6 | tetraspanin 6 |
| TYRO3 | TYRO3 protein tyrosine kinase |
| TYW1 | tRNA-yW synthesizing protein 1 homolog (S. cerevisiae) |
| UBE3D | ubiquitin protein ligase E3D |
| UBXN2B | UBX domain protein 2B |
| UFSP2 | UFM1-specific peptidase 2 |
| UHRF1BP1L | UHRF1 binding protein 1-like |
| ULK1 | unc-51 like autophagy activating kinase 1 |
| UQCC | ubiquinol-cytochrome c reductase complex chaperone |
| USP12 | ubiquitin specific peptidase 12 |
| USP38 | ubiquitin specific peptidase 38 |
| UTP20 | UTP20, small subunit processome component |
| UXS1 | UDP-glucuronate decarboxylase 1 |
| VPS18 | VPS18, CORVET/HOPS core subunit |
| WNT3A | wingless-type MMTV integration site family, member 3A |
| ZBTB2 | zinc finger and BTB domain containing 2 |
| ZDHHC20 | zinc finger, DHHC-type containing 20 |
| ZFPM2 | zinc finger protein, FOG family member 2 |
| ZGPAT | zinc finger CCCH-type and G-patch domain containing |
| ZMAT3 | zinc finger matrin-type 3 |
| ZMIZ1 | zinc finger, MIZ-type containing 1 |
| ZNF341 | zinc finger protein 341 |
| ZNF654 | zinc finger protein 654 |

Appendix Table S10. Summary of the outlier loci from Gunnison sage-grouse populations in each identified gene region. Findings from PCADAPT , the core model (“Core”), the standard covariate model including principal component 1 (“PC1”), principal component 2 (“PC2”), principal component 3 (“PC3”), spring precipitation (“Spring PPT.”), fall precipitation (“Fall PPT.”), spring maximum temperature (“Spring_Tmax.”), CTI (“CTI”), big sagebrush cover (“Big sagebrush”), dryness index (“DRI”), and RDA with associated predictor variable (“RDA Big sagebrush”, “RDA Spring PPT.”, “RDA Fall PPT.”, “RDA CTI”). The name of the gene code is listed in the left-hand column (“Gene”; see Table S9 for a list of the corresponding full gene names) followed by the name corresponding chicken chromosome where it is located (“Chromosome”), the number of total number of SNPs identified as outliers in each gene region (“# SNPs”). Impact of each SNP as predicted by SnpEff is indicated in the by counts of SNPs in gene region in the corresponding “Effect” columns.

| Gene | Chromosome | # SNPs | PCADAPT | Core | PC1 | PC2 | PC3 | Spring PPT. | Fall PPT | Spring Tmax. | Winter VPDMAX | CTI | GUR | Big sagebrush | DRI | RDA Big sagebrush | RDA Spring PPT. | RDA Fall PPT. | RDA CTI | LOW | MODERATE | MODIFIER | HIGH | | Intron | Intergenic | Downstream | Upsream | Non-synonymous | Synonymous | Splice |
| --- | --- | --- | --- | --- | --- | --- | --- | --- | --- | --- | --- | --- | --- | --- | --- | --- | --- | --- | --- | --- | --- | --- | --- | --- | --- | --- | --- | --- | --- | --- | --- |
|  |  |  | Test | | | | | | | | | | | | | | | | | Effect | | | | | | | | | | | |
| ABCC9 | 1 | 1 |  |  |  |  |  |  |  |  |  |  |  |  |  |  |  |  | * |  |  | 1 | |  | 1 |  |  |  |  |  |  |
| ABRACL | 3 | 1 |  |  |  |  |  |  |  |  |  |  |  |  |  |  |  | * |  |  |  | 1 | |  |  |  |  | 1 |  |  |  |
| ACAD11 | 2 | 1 |  |  |  |  |  |  |  |  |  |  |  |  |  |  | * |  |  |  |  | 1 | |  | 1 |  |  |  |  |  |  |
| ACOT4 | 3 | 1 | * |  |  |  |  |  |  |  |  |  |  |  |  |  |  |  |  |  |  | 1 | |  | 1 |  |  |  |  |  |  |
| ACSF3 | 11 | 1 |  |  |  |  |  |  |  |  |  |  |  |  |  |  |  | * |  |  |  | 1 | |  | 1 |  |  |  |  |  |  |
| ACSL1 | 4 | 1 |  |  |  |  |  |  |  |  |  |  |  |  |  |  |  | * |  |  |  | 1 | |  | 1 |  |  |  |  |  |  |
| ACTR2 | 3 | 2 | * |  |  |  |  |  |  |  |  |  |  |  |  |  |  |  | * |  |  | 2 | |  | 2 |  |  |  |  |  |  |
| ADAMTS20 | 1 | 5 | * | * |  |  |  |  |  |  | * |  |  | * |  |  | * |  |  |  |  | 5 | |  | 1 | 4 |  |  |  |  |  |
| ADCY5 | 7 | 1 | * |  | * |  |  |  |  |  |  |  |  |  |  |  |  |  | * |  |  | 1 | |  | 1 |  |  |  |  |  |  |
| AGO2 | 2 | 1 |  |  | * |  |  |  |  |  |  |  |  |  |  |  |  |  |  |  |  | 1 | |  | 1 |  |  |  |  |  |  |
| AGPAT9 | 4 | 2 |  | * |  |  | * |  |  |  |  | * |  |  |  |  |  |  |  |  |  | 2 | |  | 2 |  |  |  |  |  |  |
| ALMS1 | 4 | 1 |  |  |  |  |  |  |  |  |  |  |  |  |  |  |  | * |  |  |  | 1 | |  | 1 |  |  |  |  |  |  |
| ALOX5 | 6 | 1 | * |  |  |  |  |  |  |  |  |  |  |  |  |  |  |  | * |  |  | 1 | |  |  |  | 1 |  |  |  |  |
| ANKRD10 | 1 | 1 |  |  |  |  |  |  |  |  |  |  |  |  |  |  |  | * |  |  |  | 2 | |  |  | 1 |  | 1 |  |  |  |
| ANKRD13C | 8 | 4 | * | * |  |  | * |  |  |  |  |  |  |  | * |  |  | * |  |  |  | 6 | |  | 2 | 2 |  | 2 |  |  |  |
| ANKRD27 | 11 | 1 |  |  |  |  |  |  |  |  |  | * |  |  |  |  |  |  |  |  |  | 1 | |  | 1 |  |  |  |  |  |  |
| ANKRD44 | 2 | 1 |  |  |  |  |  |  |  |  |  |  | * |  |  |  |  |  |  |  |  | 1 | |  |  |  |  | 1 |  |  |  |
| ANKRD50 | 4 | 2 |  |  |  |  |  |  |  |  |  |  |  |  | * |  |  |  | * |  |  | 2 | |  | 1 | 1 |  |  |  |  |  |
| ANKS1B | 1 | 1 |  |  |  |  |  |  |  |  |  |  |  |  |  |  |  |  | * |  |  | 1 | |  | 1 |  |  |  |  |  |  |
| ANO4 | 1 | 2 |  |  |  |  |  |  |  |  |  |  |  |  |  |  | * |  |  |  |  | 2 | |  | 2 |  |  |  |  |  |  |
| APPL2 | 1 | 2 |  |  |  |  |  |  |  |  |  |  |  |  |  |  | * | * |  |  |  | 2 | |  | 1 | 1 |  |  |  |  |  |
| ARPP19 | 10 | 2 | * |  |  |  |  |  |  |  |  |  |  |  |  |  |  |  | * |  |  | 4 | |  |  | 2 | 2 |  |  |  |  |
| ASMTL | 1 | 2 |  |  |  |  |  |  |  |  |  |  |  |  | * |  | * |  |  |  |  | 2 | |  | 1 | 1 |  |  |  |  |  |
| ATXN2 | 15 | 1 |  |  |  |  |  |  |  |  |  |  |  |  |  |  |  |  | * |  |  | 1 | |  | 1 |  |  |  |  |  |  |
| AURKA | 20 | 1 |  |  |  |  |  |  |  |  |  |  |  |  |  |  | * |  |  |  |  | 1 | |  |  |  |  | 1 |  |  |  |
| AURKAIP1 | 21 | 1 |  |  |  |  |  |  |  |  |  |  |  |  | * |  |  |  |  |  |  | 1 | |  | 1 |  |  |  |  |  |  |
| BACH2 | 3 | 1 |  |  |  |  |  |  |  |  |  |  |  |  |  |  |  | * |  |  |  | 1 | |  | 1 |  |  |  |  |  |  |
| BEGAIN | 3 | 1 | * |  |  |  |  |  |  |  |  |  |  |  |  |  |  |  | * |  |  | 1 | |  | 1 |  |  |  |  |  |  |
| BEST3 | 1 | 1 |  |  |  |  |  | * |  |  |  |  |  |  | * |  |  |  |  |  |  | 1 | |  | 1 |  |  |  |  |  |  |
| BICD1 | 1 | 1 |  |  |  |  |  | * |  |  |  |  |  |  |  |  |  |  |  |  |  | 1 | |  |  |  |  | 1 |  |  |  |
| BIRC6 | 3 | 1 |  |  |  |  |  |  |  |  | * |  |  |  |  |  |  |  |  |  |  | 1 | |  | 1 |  |  |  |  |  |  |
| BMP2K | 4 | 1 |  |  |  |  |  |  |  |  |  |  |  |  |  |  |  | * |  |  |  | 1 | |  | 1 |  |  |  |  |  |  |
| BMPER | 2 | 1 | * |  |  |  |  |  |  |  |  |  |  |  |  |  |  | * |  |  |  | 1 | |  | 1 |  |  |  |  |  |  |
| BRD2 | 17 | 1 |  |  |  |  |  |  |  |  |  |  |  |  |  |  | * |  |  |  |  | 1 | |  |  |  | 1 |  |  |  |  |
| BRINP3 | 8 | 1 |  |  |  |  |  |  |  |  |  | * |  | * |  |  |  |  |  |  |  | 1 | |  | 1 |  |  |  |  |  |  |
| BRWD1 | 1 | 1 |  |  |  |  |  | * |  |  |  |  |  |  |  |  |  |  |  | 1 |  |  | |  |  |  |  |  |  |  |  |
| BUB1 | 3 | 1 |  |  |  |  |  |  |  | * |  |  |  |  |  |  |  |  |  |  |  | 1 | |  | 1 |  |  |  |  |  |  |
| C1H21ORF33 | 1 | 1 |  |  |  |  |  |  |  |  |  |  |  |  |  |  | * |  |  |  |  | 1 | |  | 1 |  |  |  |  |  |  |
| C1H3ORF38 | 1 | 1 |  |  |  |  |  |  |  |  |  |  |  |  |  |  |  |  | * |  |  | 1 | |  | 1 |  |  |  |  |  |  |
| C21H1ORF159 | 21 | 1 |  |  |  |  |  |  |  |  |  |  |  |  |  |  | * |  |  |  |  | 1 | |  | 1 |  |  |  |  |  |  |
| C4H4ORF27 | 4 | 1 | * |  |  |  |  |  |  |  |  |  |  |  |  |  |  |  |  |  |  | 1 | |  | 1 |  |  |  |  |  |  |
| C5 | 17 | 1 |  |  |  |  |  |  | * |  |  |  |  |  |  |  |  |  |  |  |  | 2 | |  |  | 1 |  | 1 |  |  |  |
| C5H11ORF58 | 5 | 1 |  |  |  |  |  |  |  |  |  |  |  |  |  |  |  |  | * |  |  | 1 | |  | 1 |  |  |  |  |  |  |
| CALCR | 2 | 1 |  |  |  | * |  |  |  |  |  |  |  |  |  |  |  |  |  |  |  | 1 | |  | 1 |  |  |  |  |  |  |
| CAPN6 | 4 | 1 |  |  |  |  | * |  |  |  |  |  |  |  |  |  |  |  |  |  |  | 1 | |  | 1 |  |  |  |  |  |  |
| CAPN7 | 2 | 2 |  |  |  |  |  |  |  |  |  |  | * |  |  |  |  |  | * |  |  | 2 | |  | 1 | 1 |  |  |  |  |  |
| CAPRIN1 | 3 | 1 |  |  |  |  |  |  | * |  |  |  |  |  |  |  |  |  |  |  |  | 1 | |  |  |  | 1 |  |  |  |  |
| CAT | 3 | 1 |  |  |  |  |  |  |  |  |  |  |  |  |  |  |  | * |  |  |  | 1 | |  |  |  | 1 |  |  |  |  |
| CC2D1B | 8 | 1 |  |  |  |  |  |  |  |  |  | * |  | * |  |  |  |  |  |  |  | 1 | |  | 1 |  |  |  |  |  |  |
| CCDC28A | 3 | 1 |  |  |  |  |  |  |  |  |  |  |  |  |  |  |  |  | * |  |  | 2 | |  |  |  |  | 2 |  |  |  |
| CCDC47 | 27 | 2 |  |  |  |  |  |  |  |  |  |  |  |  |  |  |  | * |  |  |  | 4 | |  |  | 2 |  | 2 |  |  |  |
| CD82 | 3 | 3 | * |  |  |  |  |  |  |  |  |  |  |  |  |  |  | * | * |  |  | 3 | |  |  | 2 | 1 |  |  |  |  |
| CD86 | 1 | 2 |  |  |  |  |  |  |  |  |  |  |  |  |  |  | * |  |  |  |  | 2 | |  | 2 |  |  |  |  |  |  |
| CDCP1 | 2 | 1 |  |  |  |  |  |  |  |  |  |  |  |  |  |  |  | * |  |  |  | 1 | |  | 1 |  |  |  |  |  |  |
| CENPW | 1 | 1 |  |  |  |  |  | * |  |  |  |  |  |  |  |  |  |  |  |  |  | 1 | |  | 1 |  |  |  |  |  |  |
| CEP135 | 4 | 2 |  |  |  |  |  |  |  |  |  |  | * |  |  |  |  |  | * |  |  | 2 | |  | 1 | 1 |  |  |  |  |  |
| CERS3 | 10 | 1 |  |  | * |  |  |  |  |  |  |  |  |  |  |  |  |  |  |  |  | 1 | |  |  |  | 1 |  |  |  |  |
| CHAT | 6 | 1 |  |  |  |  |  |  |  |  |  |  |  |  |  |  |  |  | * |  |  | 3 | |  |  | 1 | 2 |  |  |  |  |
| CHKA | 3 | 1 |  |  | * |  |  |  |  |  |  |  |  |  |  |  |  |  |  |  |  | 1 | |  |  |  |  | 1 |  |  |  |
| CHPT1 | 1 | 1 |  |  |  |  |  |  |  |  |  |  |  |  |  |  |  |  | * |  |  | 1 | |  | 1 |  |  |  |  |  |  |
| CLCN3 | 4 | 1 | * |  |  |  |  |  |  |  |  |  |  |  |  |  |  |  |  |  |  | 1 | |  | 1 |  |  |  |  |  |  |
| CLEC3B | 2 | 1 |  |  |  |  |  |  |  |  |  |  |  |  |  |  |  | * |  |  |  | 1 | |  |  |  | 1 |  |  |  |  |
| CMIP | 11 | 1 | * |  |  |  |  |  |  |  |  |  |  |  |  |  |  |  |  |  |  | 1 | |  | 1 |  |  |  |  |  |  |
| CMTR1 | 3 | 1 |  |  |  |  |  |  | * |  |  |  |  |  |  |  |  |  |  |  |  | 1 | |  | 1 |  |  |  |  |  |  |
| CNTRL | 17 | 1 |  |  |  |  |  |  | * |  |  |  |  |  |  |  |  |  |  |  |  | 2 | |  |  | 1 |  | 1 |  |  |  |
| COL6A3 | 2 | 3 | * | * |  |  |  |  |  |  |  |  |  | * |  |  |  |  | * |  |  | 3 | |  |  | 2 |  | 1 |  |  |  |
| CORO1C | 15 | 1 |  |  |  |  | * |  |  |  |  | * |  |  |  |  |  |  |  |  |  | 1 | |  | 1 |  |  |  |  |  |  |
| COX10 | 18 | 2 |  |  | * |  |  |  |  |  |  | * |  | * |  |  |  |  |  |  |  | 2 | |  |  | 1 |  | 1 |  |  |  |
| CRIM1 | 3 | 2 |  |  |  |  |  |  |  |  |  |  |  | * |  |  |  |  |  |  |  | 3 | |  | 1 | 1 |  | 1 |  |  |  |
| CRYAA | 1 | 1 |  |  |  |  |  |  |  |  |  |  |  |  |  |  | * |  |  |  |  | 1 | |  | 1 |  |  |  |  |  |  |
| CTH | 8 | 2 |  | * |  |  | * |  |  |  |  |  |  |  |  |  |  | * |  |  |  | 4 | |  |  | 2 |  | 2 |  |  |  |
| CYP2C23b | 6 | 3 | * |  |  |  |  |  |  |  |  |  |  |  |  |  |  |  | * |  |  | 3 | |  | 2 | 1 |  |  |  |  |  |
| CYP2R1 | 5 | 3 |  | * |  |  |  |  |  |  |  |  |  |  | * |  |  | * |  |  |  | 3 | |  | 3 |  |  |  |  |  |  |
| CYP4B1 | 8 | 1 |  |  |  |  |  |  |  |  |  |  |  |  |  |  |  | * |  |  |  | 1 | |  | 1 |  |  |  |  |  |  |
| CYP4V2 | 4 | 1 |  |  |  |  |  |  |  |  |  |  | * |  |  |  |  |  |  |  |  | 2 | |  |  | 1 | 1 |  |  |  |  |
| CYTH3 | 14 | 2 |  |  |  |  |  |  |  |  |  |  |  |  |  |  |  | * |  |  |  | 2 | |  | 2 |  |  |  |  |  |  |
| DAAM2 | 3 | 1 |  |  |  |  |  |  |  |  |  |  |  |  |  |  | * |  |  |  |  | 1 | |  | 1 |  |  |  |  |  |  |
| DDX1 | 4 | 1 |  |  | * |  |  |  |  |  |  |  |  |  |  |  |  |  | * |  |  | 1 | |  | 1 |  |  |  |  |  |  |
| DDX10 | 1 | 1 |  |  |  |  |  |  |  |  |  |  |  |  |  |  | * |  |  |  |  | 1 | |  |  |  | 1 |  |  |  |  |
| DDX42 | 27 | 2 |  |  |  |  |  |  |  |  |  |  |  |  |  |  |  | * |  |  |  | 4 | |  |  | 2 |  | 2 |  |  |  |
| DENND5A | 5 | 5 |  | * |  |  |  |  |  |  |  |  |  |  | * |  |  |  |  |  |  | 5 | |  | 5 |  |  |  |  |  |  |
| DHRS3 | 21 | 1 |  |  |  |  |  |  |  |  |  |  |  |  |  |  |  |  | * |  |  | 1 | |  | 1 |  |  |  |  |  |  |
| DNAJC13 | 2 | 1 |  |  |  |  |  |  |  |  |  |  |  |  |  |  | * |  |  |  |  | 1 | |  |  |  | 1 |  |  |  |  |
| EAF2 | 7 | 1 | * |  |  |  |  |  |  |  |  |  |  |  |  |  |  |  |  |  |  | 1 | |  |  |  | 1 |  |  |  |  |
| EHHADH | 9 | 2 |  |  | * |  |  |  |  |  |  |  |  |  |  |  |  | * |  |  |  | 2 | |  | 1 | 1 |  |  |  |  |  |
| EIF1B | 2 | 1 |  | * |  |  |  |  |  |  |  |  |  |  |  |  |  | * |  |  |  | 1 | |  |  |  | 1 |  |  |  |  |
| EPAS1 | 3 | 2 |  |  |  |  |  |  |  |  |  |  |  |  |  |  |  | * |  |  |  | 2 | |  | 2 |  |  |  |  |  |  |
| EXD2 | 3 | 1 |  |  |  |  |  |  |  |  |  |  |  |  |  |  |  | * |  |  |  | 2 | |  |  | 1 | 1 |  |  |  |  |
| EXOC1 | 4 | 2 |  |  |  |  |  |  |  |  |  |  | * |  |  |  |  |  |  |  |  | 2 | |  | 2 |  |  |  |  |  |  |
| FADS2 | 3 | 1 |  |  |  |  |  |  |  |  |  |  |  |  |  |  |  | * |  |  |  | 2 | |  |  |  | 2 |  |  |  |  |
| FAM105A | 2 | 1 |  |  |  |  |  |  |  |  |  |  |  |  |  |  |  | * |  |  |  | 1 | |  |  |  |  | 1 |  |  |  |
| FAM126A | 2 | 1 |  |  |  |  |  |  |  |  |  |  |  |  |  |  |  | * |  |  |  | 2 | |  | 1 |  | 1 |  |  |  |  |
| FAM210B | 20 | 1 |  |  |  |  |  |  |  |  |  |  |  |  |  |  | * |  |  |  |  | 1 | |  |  |  | 1 |  |  |  |  |
| FGF13 | 4 | 1 |  |  |  |  |  | * |  |  |  |  |  |  |  |  |  |  |  |  |  | 1 | |  | 1 |  |  |  |  |  |  |
| FTL | 5 | 1 |  |  |  |  |  |  |  |  |  |  |  |  |  |  |  | * |  |  |  | 1 | |  | 1 |  |  |  |  |  |  |
| FUCA2 | 3 | 1 |  |  |  |  |  |  |  |  |  |  |  |  |  |  | * |  |  |  |  | 1 | |  | 1 |  |  |  |  |  |  |
| GABRB3 | 1 | 1 |  |  |  |  |  |  |  |  |  |  |  |  |  |  |  |  | * |  |  | 1 | |  | 1 |  |  |  |  |  |  |
| GARNL3 | 17 | 2 |  |  |  |  |  |  |  |  |  |  |  |  |  |  |  | * |  |  |  | 2 | |  | 2 |  |  |  |  |  |  |
| GCOM1 | 10 | 3 |  |  |  |  |  |  |  |  |  |  |  |  |  |  |  | * | * |  |  | 3 | |  | 3 |  |  |  |  |  |  |
| GIMD1 | 4 | 1 |  |  |  |  |  |  |  |  |  |  |  |  |  | * |  |  |  |  |  | 1 | |  | 1 |  |  |  |  |  |  |
| GLRA3 | 4 | 1 |  |  |  |  |  |  |  |  |  |  |  |  |  |  | * |  |  |  |  | 1 | |  |  |  |  | 1 |  |  |  |
| GNPTAB | 1 | 2 |  |  |  |  |  |  |  |  |  |  |  |  |  |  |  | * |  |  |  | 2 | |  | 2 |  |  |  |  |  |  |
| GPM6A | 4 | 2 |  |  |  |  |  |  |  |  |  |  |  |  |  |  | * |  |  |  |  | 2 | |  | 2 |  |  |  |  |  |  |
| GRHL1 | 4 | 1 |  |  |  |  |  |  |  |  |  |  |  |  |  |  |  |  | * |  |  | 1 | |  | 1 |  |  |  |  |  |  |
| GRIN2A | 14 | 2 |  |  |  |  |  |  |  |  |  |  |  |  |  |  |  | * |  |  |  | 2 | |  | 2 |  |  |  |  |  |  |
| GRM5 | 1 | 1 |  |  |  |  |  |  |  |  |  |  |  |  |  |  |  |  | * |  |  | 1 | |  | 1 |  |  |  |  |  |  |
| GTF2H1 | 5 | 1 |  |  |  |  |  |  |  |  |  |  |  |  |  |  |  | * |  |  |  | 1 | |  | 1 |  |  |  |  |  |  |
| H2AFY | 13 | 1 |  |  |  |  |  |  |  |  |  |  |  |  |  |  | * |  |  |  |  | 2 | |  |  | 1 | 1 |  |  |  |  |
| HBP1 | 1 | 1 |  |  |  |  |  |  |  |  |  |  |  |  |  |  |  |  | * |  |  | 1 | |  | 1 |  |  |  |  |  |  |
| HOOK1 | 8 | 2 |  |  |  |  |  |  |  |  |  |  |  |  |  |  |  |  | * |  |  | 2 | |  | 2 |  |  |  |  |  |  |
| HPRT1 | 4 | 1 | * |  |  |  |  |  |  |  | * |  |  |  |  |  |  |  |  |  |  | 1 | |  |  |  | 1 |  |  |  |  |
| HSD17B12 | 3 | 1 |  |  |  |  |  |  |  |  |  |  |  |  |  |  |  | * |  |  |  | 1 | |  | 1 |  |  |  |  |  |  |
| HSP90B1 | 1 | 1 |  |  |  |  |  |  |  |  |  |  |  |  |  |  |  | * |  |  |  | 1 | |  | 1 |  |  |  |  |  |  |
| IL18RAP | 1 | 1 |  |  |  |  |  |  |  |  |  |  |  |  |  |  |  |  | * |  |  | 1 | |  | 1 |  |  |  |  |  |  |
| IL1R2 | 1 | 1 |  |  |  |  |  |  |  |  |  |  |  |  |  |  |  |  | * |  |  | 1 | |  |  |  |  | 1 |  |  |  |
| INF2 | 3 | 1 |  |  |  |  |  |  |  |  |  |  |  |  |  |  | * |  |  |  |  | 1 | |  | 1 |  |  |  |  |  |  |
| INPP5D | 9 | 1 |  |  |  |  |  |  |  |  |  |  |  |  |  |  | * |  |  |  |  | 1 | |  | 1 |  |  |  |  |  |  |
| INPPL1 | 4 | 1 |  |  |  |  |  |  |  |  |  |  |  |  |  |  |  | * |  |  |  | 1 | |  |  |  | 1 |  |  |  |  |
| IQCE | 14 | 1 |  | * |  |  |  |  |  |  |  |  |  |  |  |  |  | * |  |  |  | 1 | |  |  |  |  | 1 |  |  |  |
| IRAK4 | 1 | 3 | * |  |  |  |  | * |  |  | * |  |  |  |  |  | * |  |  |  |  | 3 | |  | 3 |  |  |  |  |  |  |
| IRF2 | 4 | 2 |  |  |  |  |  |  |  |  |  |  |  | * |  |  | * |  | * |  |  | 2 | |  |  |  | 2 |  |  |  |  |
| KCNMB4 | 1 | 1 |  |  |  |  | * |  |  |  |  |  |  |  |  |  |  |  |  |  |  | 1 | |  | 1 |  |  |  |  |  |  |
| KIAA1210 | 4 | 1 | * | * |  |  |  |  |  |  |  |  |  |  |  |  |  | * |  |  |  | 1 | |  | 1 |  |  |  |  |  |  |
| KIAA1549L | 5 | 2 |  |  |  |  |  |  |  |  |  |  |  |  |  |  | * |  |  |  |  | 2 | |  |  |  | 2 |  |  |  |  |
| KIF13A | 2 | 1 |  |  |  |  |  |  |  |  |  |  |  |  |  |  |  | * |  |  |  | 1 | |  | 1 |  |  |  |  |  |  |
| KIF18A | 6 | 1 |  |  |  |  |  |  |  |  | * |  |  |  |  |  |  |  |  |  |  | 1 | |  |  |  |  | 1 |  |  |  |
| KIF23 | 10 | 1 |  |  |  |  |  | * |  |  |  |  |  |  |  |  |  |  |  |  |  | 1 | |  |  |  |  | 1 |  |  |  |
| KRT20 | 27 | 1 |  |  |  |  |  |  |  |  |  |  |  |  |  |  |  | * |  |  |  | 2 | |  |  | 1 |  | 1 |  |  |  |
| LAMA5 | 20 | 2 | * |  |  |  |  |  |  |  |  |  |  |  |  |  |  |  |  |  |  | 2 | |  | 2 |  |  |  |  |  |  |
| LAMP3 | 9 | 1 |  |  |  |  |  |  |  |  |  |  |  |  |  |  | * |  |  |  |  | 1 | |  | 1 |  |  |  |  |  |  |
| LGALSL | 3 | 3 | * | * |  |  |  |  |  |  |  |  |  |  |  |  |  |  | * |  |  | 3 | |  |  | 1 | 2 |  |  |  |  |
| LOC101751732 | 8 | 2 |  |  |  |  | * |  |  |  |  | * |  |  |  |  |  |  |  |  |  | 2 | |  | 2 |  |  |  |  |  |  |
| LOC419830 | 26 | 2 |  |  |  |  |  |  |  | * |  |  |  |  |  |  |  |  |  |  |  | 4 | |  |  | 2 | 2 |  |  |  |  |
| LOC424998 | 9 | 1 |  |  |  |  |  |  |  |  |  |  |  |  |  |  |  |  | * |  |  | 1 | |  | 1 |  |  |  |  |  |  |
| LOC427545 | 11 | 1 |  |  |  |  |  |  |  |  |  |  |  |  |  |  |  | * |  |  |  | 2 | |  |  | 1 |  | 1 |  |  |  |
| LOC768589 | 2 | 1 |  |  |  |  |  | * |  |  |  |  |  |  |  |  |  |  |  |  |  | 1 | |  | 1 |  |  |  |  |  |  |
| LOC771638 | 14 | 1 |  |  |  |  |  |  |  |  |  |  |  |  |  |  |  | * |  |  |  | 2 | |  |  | 1 | 1 |  |  |  |  |
| LRCH2 | 4 | 1 |  |  |  |  |  |  |  |  |  |  |  |  |  |  | * |  |  |  |  | 1 | |  | 1 |  |  |  |  |  |  |
| LRIT2 | 6 | 2 | * |  | * |  |  |  |  |  |  |  |  |  |  |  |  |  | * |  |  | 2 | |  | 2 |  |  |  |  |  |  |
| LRP2BP | 4 | 1 |  |  |  |  |  |  |  |  |  |  |  |  |  |  |  | * |  |  |  | 1 | |  |  |  |  | 1 |  |  |  |
| LRP4 | 3 | 1 |  |  |  |  |  |  |  |  |  |  |  |  |  |  |  | * |  |  |  | 2 | |  |  | 1 |  | 1 |  |  |  |
| LYVE1 | 5 | 2 |  |  |  |  |  |  |  |  |  |  |  |  |  |  | * |  |  | 1 |  | 1 | |  | 1 |  |  |  |  |  |  |
| MAP2 | 2 | 1 |  |  |  |  |  |  |  |  |  |  |  |  |  |  |  | * |  |  |  | 1 | |  | 1 |  |  |  |  |  |  |
| MAP3K3 | 27 | 1 |  |  |  |  |  |  |  |  |  |  |  |  |  |  |  | * |  |  |  | 1 | |  | 1 |  |  |  |  |  |  |
| MAP7D2 | 1 | 1 |  |  |  |  |  |  |  |  |  |  | * |  |  |  |  |  |  |  |  | 1 | |  | 1 |  |  |  |  |  |  |
| MARCH1 | 4 | 2 | * |  |  |  |  |  |  |  |  |  |  |  |  |  |  |  | * |  |  | 2 | |  | 2 |  |  |  |  |  |  |
| MARCH8 | 6 | 2 | * |  |  |  |  |  |  |  |  |  |  |  |  |  |  |  | * |  |  | 2 | |  | 2 |  |  |  |  |  |  |
| MAT1A | 6 | 1 |  |  |  |  | * |  |  |  |  |  |  |  |  |  |  |  |  |  |  | 1 | |  | 1 |  |  |  |  |  |  |
| MCM4 | 1 | 1 | * | * |  |  |  |  |  |  |  |  |  |  |  |  |  |  |  |  |  | 1 | |  |  |  |  | 1 |  |  |  |
| MEP1A | 3 | 1 |  | * |  |  |  | * |  |  |  |  |  |  |  |  |  |  |  |  |  | 2 | |  |  | 1 | 1 |  |  |  |  |
| MICALL2 | 14 | 1 |  |  |  |  |  |  |  |  |  |  |  |  |  |  |  |  | * |  |  | 1 | |  |  |  |  | 1 |  |  |  |
| MMP16 | 2 | 2 |  |  |  |  |  |  |  |  |  |  |  |  |  |  |  | * | * |  |  | 2 | |  | 2 |  |  |  |  |  |  |
| MORF4L1 | 10 | 1 |  |  | * |  |  |  |  | * |  |  |  |  |  |  |  |  |  |  | 1 |  | |  |  |  |  |  | 1 |  |  |
| MSN | 4 | 1 |  |  |  |  |  |  |  |  |  |  |  |  |  | * |  |  |  |  |  | 3 | |  |  | 1 | 2 |  |  |  |  |
| MTF1 | 23 | 1 |  |  |  |  |  |  |  |  |  |  |  |  |  |  |  | * |  |  |  | 1 | |  | 1 |  |  |  |  |  |  |
| MYBPC3 | 3 | 1 |  |  | * |  |  |  |  |  |  |  |  |  |  |  |  |  |  |  |  | 1 | |  | 1 |  |  |  |  |  |  |
| MYLIP | 2 | 2 |  |  |  |  |  |  |  |  |  |  |  |  |  |  | * | * |  | 1 |  | 1 | |  | 1 |  |  |  |  | 1 |  |
| MYO1B | 2 | 4 |  |  |  |  |  |  |  |  |  |  |  |  |  |  |  | * | * |  |  | 5 | |  | 2 | 2 | 1 |  |  |  |  |
| MYO1H | 15 | 1 | * | * |  |  |  | * |  |  |  |  |  |  |  |  |  |  |  |  |  | 1 | |  | 1 |  |  |  |  |  |  |
| NAALAD2 | 1 | 2 |  |  |  |  |  |  |  |  |  |  |  |  |  | * |  |  | * |  |  | 2 | |  | 2 |  |  |  |  |  |  |
| NAB1 | 2 | 5 |  |  |  |  |  |  |  |  |  |  |  |  |  |  |  | * |  |  |  | 5 | |  | 3 | 2 |  |  |  |  |  |
| NADSYN1 | 5 | 1 |  |  |  |  |  |  |  |  |  |  |  |  |  |  |  |  | * |  |  | 1 | |  | 1 |  |  |  |  |  |  |
| NAP1L4 | 5 | 1 |  |  |  |  |  |  |  |  |  |  |  |  | * |  |  |  |  |  |  | 2 | |  |  | 1 | 1 |  |  |  |  |
| NBAS | 4 | 1 | * | * |  |  |  |  |  |  |  |  |  |  |  |  |  |  | * |  |  | 1 | |  | 1 |  |  |  |  |  |  |
| NCKAP1 | 2 | 3 |  |  |  |  |  |  |  |  |  |  |  |  |  |  |  | * |  |  |  | 3 | |  | 3 |  |  |  |  |  |  |
| NDUFS3 | 3 | 1 |  |  |  |  |  |  |  |  |  |  |  |  |  |  | * |  |  |  |  | 2 | |  |  | 1 | 1 |  |  |  |  |
| NMBR | 3 | 1 |  |  |  |  |  |  |  |  |  |  |  |  | * |  |  |  |  |  |  | 1 | |  | 1 |  |  |  |  |  |  |
| NSRP1 | 19 | 1 |  |  |  |  |  |  |  |  |  |  |  |  |  |  |  | * |  |  |  | 1 | |  | 1 |  |  |  |  |  |  |
| NTPCR | 3 | 1 | * |  |  |  |  |  |  |  |  |  |  |  |  |  |  |  |  |  |  | 1 | |  |  |  |  | 1 |  |  |  |
| NUCKS1 | 26 | 1 |  |  |  |  |  |  |  |  |  |  |  |  |  |  |  | * |  |  |  | 2 | |  |  | 1 | 1 |  |  |  |  |
| NUP205 | 1 | 1 |  |  |  |  |  |  |  |  |  |  |  |  |  |  | * |  |  |  |  | 1 | |  | 1 |  |  |  |  |  |  |
| OPN4-1 | 4 | 1 |  |  |  |  |  |  |  |  |  |  |  |  |  |  |  |  | * |  |  | 1 | |  | 1 |  |  |  |  |  |  |
| ORC1 | 8 | 1 |  |  |  |  |  |  |  |  |  | * |  | * |  |  |  |  |  |  |  | 1 | |  |  |  | 1 |  |  |  |  |
| P2RY4 | 4 | 1 |  |  |  |  |  |  |  |  |  |  |  |  |  |  |  | * |  |  |  | 1 | |  |  |  |  | 1 |  |  |  |
| PAK2 | 9 | 1 |  |  |  |  |  |  |  |  |  |  |  |  | * |  |  |  |  |  |  | 1 | |  | 1 |  |  |  |  |  |  |
| PALLD | 4 | 1 | * | * |  |  |  |  |  |  |  |  |  |  |  |  |  |  | * |  |  | 2 | |  |  | 1 |  | 1 |  |  |  |
| PARVA | 5 | 1 |  |  |  |  |  |  |  |  |  |  |  |  | * |  |  |  |  |  |  | 1 | |  | 1 |  |  |  |  |  |  |
| PCDH12 | 13 | 1 |  |  |  |  |  |  |  |  |  |  |  |  |  |  | * |  |  |  |  | 1 | |  |  |  | 1 |  |  |  |  |
| PCDH9 | 1 | 10 | * |  | * |  |  |  |  |  |  |  | * |  |  |  |  |  |  |  |  | 10 | |  | 9 | 1 |  |  |  |  |  |
| PDE3B | 5 | 1 |  |  |  |  | * |  |  |  |  | * |  |  |  |  |  |  |  |  |  | 2 | |  |  | 1 |  | 1 |  |  |  |
| PHACTR1 | 2 | 1 |  | * |  |  |  |  | * |  | * |  |  |  |  |  | * |  |  |  |  | 1 | |  | 1 |  |  |  |  |  |  |
| PHKA2 | 1 | 1 |  |  |  |  |  |  |  |  |  |  |  |  |  |  | * |  |  |  |  | 4 | |  |  | 2 | 1 | 1 |  |  |  |
| PI4K2B | 4 | 1 |  |  |  |  |  |  |  |  |  |  |  |  |  |  |  |  | * |  |  | 1 | |  | 1 |  |  |  |  |  |  |
| PIK3CA | 9 | 2 |  |  |  |  |  |  |  |  |  |  |  |  |  |  |  | * |  |  |  | 2 | |  | 1 |  |  | 1 |  |  |  |
| PKP2 | 1 | 1 | * |  |  |  |  |  |  |  |  |  |  | * |  |  |  |  |  |  |  | 1 | |  | 1 |  |  |  |  |  |  |
| PLCB1 | 3 | 1 | * |  |  |  |  |  |  |  |  |  |  |  |  |  |  |  |  |  |  | 1 | |  | 1 |  |  |  |  |  |  |
| PLEKHA7 | 5 | 1 |  |  |  |  |  |  |  |  |  |  |  |  |  |  |  |  | * |  |  | 1 | |  | 1 |  |  |  |  |  |  |
| PLEKHG4 | 11 | 1 |  |  |  |  |  |  |  |  |  |  |  |  |  |  |  |  | * |  |  | 1 | |  | 1 |  |  |  |  |  |  |
| PLIN3 | 20 | 1 |  | * |  |  |  |  |  |  |  |  |  |  |  |  | * |  |  |  |  | 2 | |  |  |  | 2 |  |  |  |  |
| POLR3B | 1 | 3 |  |  |  |  |  |  |  |  |  |  |  |  |  |  | * | * |  |  |  | 3 | |  | 1 |  | 2 |  |  |  |  |
| PPARA | 1 | 1 |  |  |  |  |  |  | * |  |  |  |  |  |  |  |  |  |  |  |  | 1 | |  | 1 |  |  |  |  |  |  |
| PPP1R12A | 1 | 2 |  |  |  |  |  |  |  |  |  |  |  |  |  | * |  |  | * |  |  | 2 | |  | 1 | 1 |  |  |  |  |  |
| PRKAR1B | 14 | 1 |  |  |  | * |  |  |  |  |  |  |  |  |  |  |  |  |  |  |  | 1 | |  | 1 |  |  |  |  |  |  |
| PRKDC | 1 | 1 | * | * |  |  |  |  |  |  |  |  |  |  |  |  |  |  |  |  | 1 |  | |  |  |  |  |  | 1 |  |  |
| PRRG1 | 1 | 1 |  |  |  |  |  |  |  |  |  |  |  | * |  |  |  |  |  |  |  | 1 | |  | 1 |  |  |  |  |  |  |
| PSMG4 | 2 | 1 |  |  |  |  |  |  |  |  |  |  |  |  |  |  | * |  |  |  |  | 1 | |  | 1 |  |  |  |  |  |  |
| PTPLA | 2 | 1 |  |  |  |  |  |  |  |  |  |  |  |  |  |  | * |  |  |  |  | 1 | |  | 1 |  |  |  |  |  |  |
| PTPRT | 20 | 3 |  | * |  |  |  |  |  |  |  |  |  |  |  |  | * |  |  |  |  | 3 | |  |  |  | 3 |  |  |  |  |
| PUM2 | 3 | 1 |  |  |  |  |  | * |  |  |  |  |  |  |  |  |  |  |  |  |  | 1 | |  | 1 |  |  |  |  |  |  |
| PUS7L | 1 | 4 | * |  |  |  |  | * |  |  | * |  |  |  |  |  | * |  |  |  |  | 4 | |  |  | 1 |  | 3 |  |  |  |
| RAB11FIP4 | 18 | 1 |  |  |  |  |  |  |  |  |  |  |  |  |  |  |  |  | * |  |  | 1 | |  | 1 |  |  |  |  |  |  |
| RAP1GDS1L | 6 | 2 |  |  |  |  | * |  |  |  |  | * |  |  |  |  | * |  |  |  |  | 2 | |  | 1 | 1 |  |  |  |  |  |
| RELN | 1 | 2 | * |  |  |  |  |  |  |  |  |  |  |  |  |  |  |  |  |  |  | 2 | |  | 2 |  |  |  |  |  |  |
| RFC1 | 4 | 1 |  |  |  |  |  |  |  |  |  |  | * |  |  |  |  |  |  |  |  | 1 | |  |  |  |  | 1 |  |  |  |
| RIC8B | 1 | 1 |  | * |  |  |  |  | * |  | * |  |  |  |  |  |  |  |  |  |  | 1 | |  | 1 |  |  |  |  |  |  |
| RNF149 | 1 | 1 | * | * |  |  | * |  |  |  |  |  |  |  |  |  |  |  |  |  |  | 1 | |  | 1 |  |  |  |  |  |  |
| RNF216 | 14 | 1 |  |  |  |  |  |  |  |  |  |  |  |  |  |  |  |  | * |  |  | 1 | |  | 1 |  |  |  |  |  |  |
| RPIA | 4 | 1 |  |  |  |  |  |  |  |  |  |  |  |  |  |  |  |  | * |  |  | 1 | |  |  |  | 1 |  |  |  |  |
| RPS6KC1 | 3 | 4 |  |  |  |  |  |  |  |  |  |  |  |  |  |  | * |  | * |  |  | 4 | |  | 2 | 2 |  |  |  |  |  |
| RPSA | 2 | 2 |  |  |  |  |  |  |  |  |  |  |  |  |  |  |  | * |  |  |  | 2 | |  | 2 |  |  |  |  |  |  |
| RRP1B | 1 | 1 |  |  |  |  |  |  |  |  |  |  |  |  |  |  |  |  | * |  |  | 1 | |  | 1 |  |  |  |  |  |  |
| RUFY3 | 4 | 1 |  |  |  |  |  |  |  |  |  |  |  |  |  |  |  | * |  |  |  | 1 | |  | 1 |  |  |  |  |  |  |
| RWDD1 | 1 | 1 |  |  |  |  |  |  |  |  |  |  |  | * |  |  |  |  |  |  |  | 1 | |  | 1 |  |  |  |  |  |  |
| RWDD4 | 4 | 2 |  | * |  |  |  |  |  |  |  |  |  |  | * |  |  | * |  |  |  | 2 | |  | 2 |  |  |  |  |  |  |
| SCCPDH | 3 | 1 |  |  |  |  |  |  |  |  |  |  |  |  |  |  | * |  |  |  |  | 1 | |  | 1 |  |  |  |  |  |  |
| SDK1 | 14 | 1 | * |  |  |  |  |  |  |  |  |  |  |  |  |  |  | * |  |  |  | 1 | |  | 1 |  |  |  |  |  |  |
| SDR42E2 | 14 | 1 |  |  |  |  |  |  |  |  |  |  |  |  |  |  |  | * |  |  |  | 2 | |  |  | 1 |  | 1 |  |  |  |
| SEC16A | 17 | 1 |  | * | * |  |  |  |  |  |  |  |  |  |  |  |  |  | * |  |  | 1 | |  |  |  |  | 1 |  |  |  |
| SEC22A | 7 | 1 | * |  | * |  |  |  |  |  |  |  |  |  |  |  |  |  | * |  |  | 1 | |  | 1 |  |  |  |  |  |  |
| SEPSECS | 4 | 1 |  |  |  |  |  |  |  |  |  |  |  |  |  |  |  |  | * |  |  | 1 | |  | 1 |  |  |  |  |  |  |
| SERAC1 | 3 | 2 |  |  |  |  |  |  |  |  |  |  |  |  |  |  |  | * |  |  |  | 2 | |  | 2 |  |  |  |  |  |  |
| SETX | 17 | 1 |  |  |  |  |  |  |  |  |  |  |  |  | * |  |  |  |  |  |  | 2 | |  |  | 1 |  | 1 |  |  |  |
| SLC12A4 | 11 | 2 |  |  |  |  |  |  |  |  |  |  |  |  |  |  | * | * |  |  |  | 2 | |  | 2 |  |  |  |  |  |  |
| SLC13A5 | 19 | 1 |  | * |  |  |  |  |  |  |  |  |  |  |  |  |  |  |  |  |  | 1 | |  | 1 |  |  |  |  |  |  |
| SLC25A22 | 3 | 1 |  |  |  |  | * |  |  |  |  |  |  |  |  |  |  |  |  |  |  | 1 | |  | 1 |  |  |  |  |  |  |
| SLC2A8 | 17 | 3 |  |  |  |  |  |  | * |  |  |  |  |  |  |  |  | * |  |  |  | 3 | |  | 2 | 1 |  |  |  |  |  |
| SLC39A11 | 18 | 1 |  |  |  |  | * |  |  |  |  |  |  |  |  |  |  |  |  |  |  | 2 | |  |  | 1 |  | 1 |  |  |  |
| SLC9A4 | 1 | 1 |  |  |  |  |  |  |  |  |  |  |  |  |  |  |  |  | * |  |  | 1 | |  |  |  |  | 1 |  |  |  |
| SMPD4 | 15 | 1 |  |  |  |  |  |  |  |  |  |  |  |  |  |  |  | * |  |  |  | 1 | |  | 1 |  |  |  |  |  |  |
| SMURF1 | 14 | 1 |  |  |  |  |  |  |  |  |  |  |  |  |  |  |  | * |  |  |  | 1 | |  | 1 |  |  |  |  |  |  |
| SNAP47 | 2 | 2 |  |  |  |  |  |  | * |  | * |  |  |  |  |  |  |  |  |  |  | 2 | |  | 2 |  |  |  |  |  |  |
| SPAG9 | 18 | 1 |  |  |  |  | * |  |  |  |  |  |  |  |  |  |  |  |  |  |  | 1 | |  | 1 |  |  |  |  |  |  |
| SPATA17 | 3 | 1 |  |  |  |  |  |  |  |  |  |  |  |  |  |  |  |  | * |  |  | 1 | |  | 1 |  |  |  |  |  |  |
| SPRYD7 | 1 | 1 |  |  |  |  |  |  |  |  | * |  |  |  |  |  |  |  |  |  |  | 1 | |  |  |  | 1 |  |  |  |  |
| SRD5A3 | 4 | 1 |  |  |  |  |  |  |  |  |  |  | * |  |  |  |  |  |  |  |  | 2 | |  |  | 1 |  | 1 |  |  |  |
| SRPX2 | 4 | 1 |  |  |  |  |  |  |  |  |  |  |  |  |  |  | * |  |  |  |  | 1 | |  | 1 |  |  |  |  |  |  |
| SRRL | 6 | 3 |  | * |  |  | * |  |  |  |  | * |  |  |  |  |  |  |  |  |  | 3 | |  | 3 |  |  |  |  |  |  |
| ST6GAL2 | 1 | 1 |  |  |  |  |  |  |  |  |  |  |  |  |  |  |  | * |  |  |  | 1 | |  | 1 |  |  |  |  |  |  |
| STAG1 | 9 | 1 |  |  |  |  |  | * |  |  |  |  |  |  |  |  |  |  |  |  |  | 1 | |  | 1 |  |  |  |  |  |  |
| SYNE2 | 5 | 1 |  |  |  |  | * |  |  |  |  |  |  |  |  |  |  |  |  |  |  | 1 | |  | 1 |  |  |  |  |  |  |
| SYNE3 | 3 | 1 |  |  |  |  | * |  |  |  |  |  |  |  |  |  |  |  |  |  |  | 1 | |  | 1 |  |  |  |  |  |  |
| SYTL4 | 4 | 1 |  |  |  |  |  |  |  |  |  |  |  |  |  |  | * |  |  |  |  | 1 | |  |  |  | 1 |  |  |  |  |
| TAB2 | 3 | 1 |  |  |  |  |  |  |  |  |  |  |  |  |  |  |  | * |  |  |  | 1 | |  | 1 |  |  |  |  |  |  |
| TBC1D19 | 4 | 1 | * |  |  |  |  |  |  |  |  |  |  |  |  |  |  |  | * |  |  | 1 | |  |  |  |  | 1 |  |  |  |
| TBC1D9 | 4 | 2 |  | * |  |  |  |  |  |  |  |  |  |  | * |  |  | * |  |  |  | 2 | |  | 2 |  |  |  |  |  |  |
| TBPL1 | 3 | 1 |  |  |  |  |  |  |  | * |  |  |  |  |  |  |  |  |  |  |  | 1 | |  | 1 |  |  |  |  |  |  |
| TCEANC | 1 | 1 |  |  |  |  |  |  |  |  |  |  |  |  |  |  | * |  |  |  |  | 2 | |  |  | 1 | 1 |  |  |  |  |
| TCF21 | 3 | 2 | * |  |  |  |  |  |  |  |  |  |  |  |  |  | * |  |  |  |  | 2 | |  |  | 1 |  | 1 |  |  |  |
| TCF7 | 13 | 1 |  |  |  |  |  |  |  |  |  |  |  |  |  |  |  | * |  |  |  | 1 | |  | 1 |  |  |  |  |  |  |
| TFAP2D | 3 | 2 | * |  |  |  |  |  |  |  |  |  |  |  |  |  | * |  |  |  |  | 2 | |  | 1 | 1 |  |  |  |  |  |
| TGFBRAP1 | 1 | 1 |  |  |  |  |  |  |  |  |  |  |  |  |  |  |  | * |  |  |  | 1 | |  | 1 |  |  |  |  |  |  |
| TH | 5 | 1 |  |  |  |  |  |  |  |  |  |  | * |  |  |  |  |  |  |  |  | 1 | |  | 1 |  |  |  |  |  |  |
| TJAP1 | 3 | 1 |  |  |  |  |  |  |  |  |  |  |  |  |  |  |  | * |  |  |  | 1 | |  | 1 |  |  |  |  |  |  |
| TMA16 | 4 | 1 |  |  |  |  |  |  |  |  |  |  |  |  |  |  |  |  | * |  |  | 1 | |  |  |  | 1 |  |  |  |  |
| TMEM242 | 3 | 1 |  |  |  |  |  |  |  |  |  |  | * |  |  |  |  |  |  |  |  | 1 | |  | 1 |  |  |  |  |  |  |
| TMEM59 | 8 | 2 | * |  |  |  |  |  |  |  |  |  |  | * |  |  |  |  |  |  |  | 2 | |  | 2 |  |  |  |  |  |  |
| TMEM68 | 1 | 1 |  |  |  | * |  |  |  |  |  |  |  |  |  |  |  |  |  |  |  | 1 | |  |  |  |  | 1 |  |  |  |
| TNFSF13B | 1 | 2 |  | * |  |  |  |  |  |  |  |  |  |  |  |  | * |  | * |  |  | 2 | |  | 1 | 1 |  |  |  |  |  |
| TNRC18 | 14 | 1 |  |  | * |  |  |  |  |  |  |  |  |  |  |  |  |  |  |  |  | 1 | |  | 1 |  |  |  |  |  |  |
| TPCN3 | 3 | 1 |  |  |  |  |  |  |  | * |  |  |  |  |  |  |  |  |  |  |  | 1 | |  |  |  | 1 |  |  |  |  |
| TPP2 | 1 | 1 |  |  |  |  |  |  |  |  |  |  |  |  |  |  | * |  |  |  |  | 1 | |  | 1 |  |  |  |  |  |  |
| TRAPPC10 | 1 | 1 |  |  | * |  |  |  |  | * |  |  |  |  |  |  |  |  |  |  |  | 1 | |  | 1 |  |  |  |  |  |  |
| TRAPPC11 | 4 | 1 |  | * |  |  |  |  |  |  |  |  |  |  | * |  |  |  |  |  |  | 1 | |  |  |  |  | 1 |  |  |  |
| TRPM8 | 2 | 1 | * |  |  |  |  |  |  |  |  |  |  |  |  |  |  |  |  |  |  | 2 | |  | 1 |  | 1 |  |  |  |  |
| TSC1 | 17 | 1 |  |  |  |  |  |  |  |  |  |  |  |  |  |  |  |  | * |  |  | 1 | |  | 1 |  |  |  |  |  |  |
| TSNAXIP1 | 11 | 1 |  |  |  |  |  |  |  |  |  |  |  |  |  |  |  |  | * |  |  | 1 | |  | 1 |  |  |  |  |  |  |
| TSPAN6 | 4 | 1 |  |  |  |  |  |  |  |  |  |  |  |  |  |  | * |  |  |  |  | 1 | |  |  |  |  | 1 |  |  |  |
| TYRO3 | 3 | 1 |  |  |  |  |  |  |  |  |  |  |  |  |  |  |  |  | * |  |  | 1 | |  | 1 |  |  |  |  |  |  |
| TYW1 | 19 | 1 |  |  | * |  |  |  |  | * |  |  |  |  |  |  |  |  |  |  |  | 1 | |  | 1 |  |  |  |  |  |  |
| UBE3D | 3 | 1 |  |  |  |  |  |  |  |  |  |  |  |  |  |  | * |  |  |  |  | 1 | |  | 1 |  |  |  |  |  |  |
| UBXN2B | 1 | 4 | * |  |  |  |  |  |  |  |  |  |  |  |  |  |  | * |  |  |  | 4 | |  | 3 | 1 |  |  |  |  |  |
| UFSP2 | 4 | 1 |  |  |  |  |  |  |  |  |  |  |  |  |  |  |  | * |  |  |  | 1 | |  | 1 |  |  |  |  |  |  |
| UHRF1BP1L | 1 | 1 |  |  |  |  |  |  |  |  |  |  |  |  |  |  |  |  | * |  |  | 1 | |  | 1 |  |  |  |  |  |  |
| ULK1 | 15 | 1 |  |  |  |  |  |  |  |  |  |  |  |  |  |  |  | * |  |  |  | 1 | |  | 1 |  |  |  |  |  |  |
| UQCC | 20 | 3 | * |  |  |  | * |  |  |  |  |  |  |  |  |  |  |  |  |  |  | 3 | |  | 3 |  |  |  |  |  |  |
| USP12 | 1 | 2 |  | * |  |  |  |  |  |  |  |  |  |  |  |  |  | * | * |  |  | 2 | |  | 1 | 1 |  |  |  |  |  |
| USP38 | 4 | 3 |  |  |  |  |  |  |  |  |  |  |  |  |  |  | * | * |  |  |  | 3 | |  | 2 | 1 |  |  |  |  |  |
| UTP20 | 1 | 3 | * |  |  |  |  |  |  |  |  |  |  |  |  |  | * |  |  |  |  | 3 | |  | 3 |  |  |  |  |  |  |
| UXS1 | 1 | 1 |  | * |  |  |  |  |  |  |  |  |  |  |  |  |  |  |  |  |  | 1 | |  | 1 |  |  |  |  |  |  |
| VPS18 | 3 | 3 |  |  |  |  |  |  |  |  |  |  | * |  |  |  | * |  | * |  |  | 4 | |  |  | 3 |  | 1 |  |  |  |
| WNT3A | 2 | 1 | * |  |  |  |  |  |  |  |  |  |  |  |  |  |  |  |  |  |  | 1 | |  |  |  | 1 |  |  |  |  |
| ZBTB2 | 3 | 1 |  |  |  |  |  |  |  |  |  |  |  |  |  |  | * |  |  |  |  |  | | 1 |  |  |  |  | 1 |  |  |
| ZDHHC20 | 1 | 1 |  |  |  |  |  |  |  |  |  |  |  |  |  |  |  | * |  |  |  | 1 | |  | 1 |  |  |  |  |  |  |
| ZFPM2 | 2 | 1 |  |  |  |  |  |  | * |  |  |  |  |  |  |  |  |  |  |  |  | 1 | |  | 1 |  |  |  |  |  |  |
| ZGPAT | 20 | 1 | * |  |  |  |  |  |  |  |  |  |  |  |  |  |  |  | * | 1 |  |  | |  |  |  |  |  |  | 1 |  |
| ZMAT3 | 9 | 1 |  |  |  |  |  |  |  |  |  |  |  |  |  |  |  | * |  |  |  | 1 | |  | 1 |  |  |  |  |  |  |
| ZMIZ1 | 6 | 3 | * |  | * |  |  |  |  |  |  | * |  | * |  |  |  |  | * |  |  | 4 | |  |  | 3 |  | 1 |  |  |  |
| ZNF341 | 20 | 2 | * | * |  |  |  |  | * |  | * |  |  |  |  |  | * |  |  |  |  | 2 | |  | 2 |  |  |  |  |  |  |
| ZNF654 | 1 | 1 |  |  |  |  |  |  |  |  |  |  |  |  |  |  |  |  | * |  |  | 1 | |  |  |  | 1 |  |  |  |  |

Appendix Figure S11. Three-dimensional plots of the first three principal components for each PCA analysis on SNPs under divergent selection from different methods and covariates. Each point represents an individual color coded by the population where the sample was collected: Cimarron = red, Crawford = blue, Dove Creek = green, Gunnison Basin = purple, Piñon Mesa = orange, San Miguel = yellow. Percentage of variation accounted for by each PC is labeled in parentheses on each axis.


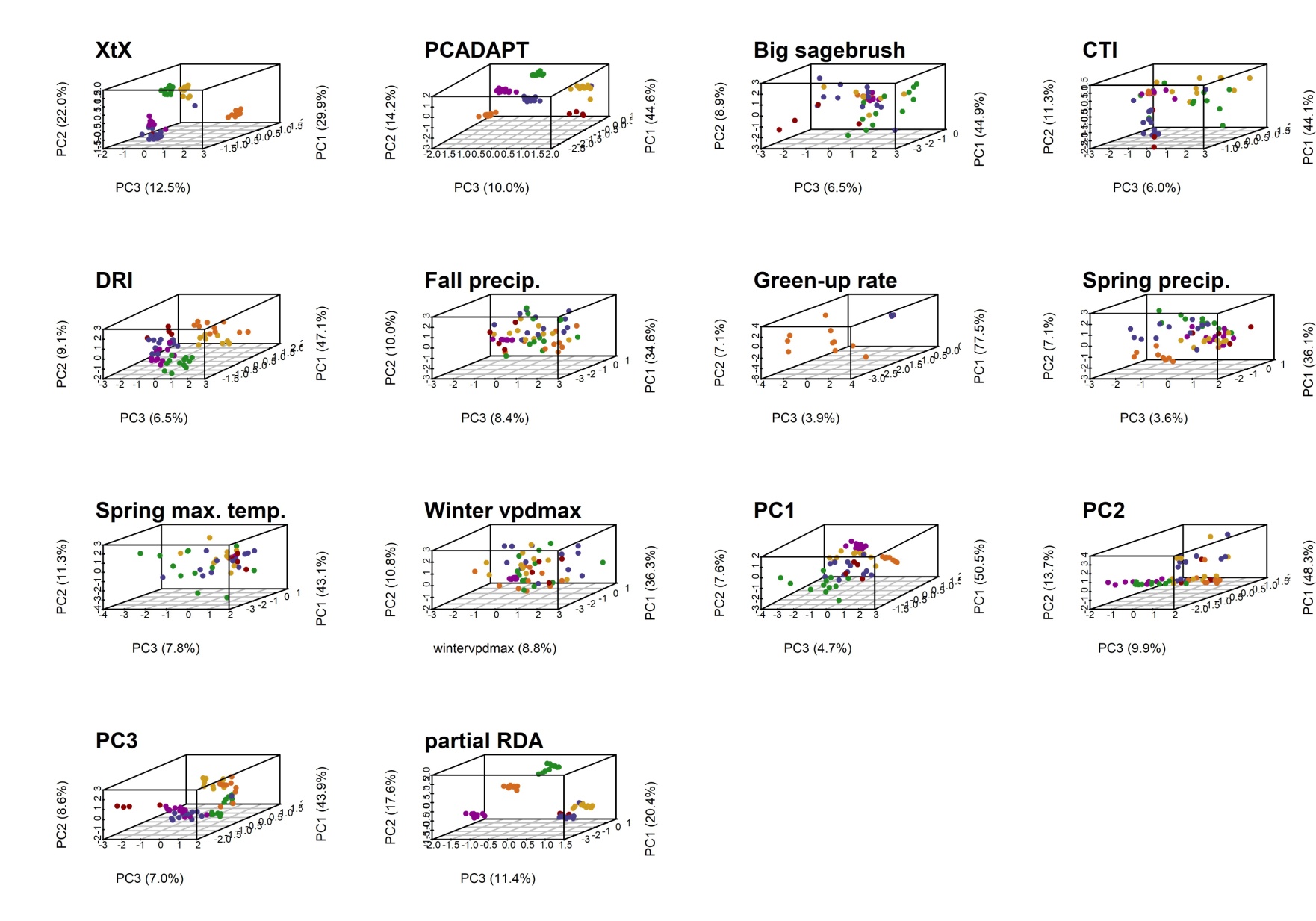


Appendix Table S13. West Nile virus detection in counties supporting Gunnison sage-grouse populations. Detections = total number of organisms (animals and mosquitos) detected with WNV from 2006-2017. Colorado data source: <https://www.colorado.gov/pacific/cdphe/west-nile-virus-data>; Utah data source: <http://health.utah.gov/epi/diseases/WNV/surveillance>.

| County | Population | Detections |
| --- | --- | --- |
| Montrose | Cimarron & Crawford | 5 |
| Delta | Crawford | 27 |
| Dolores | Dove Creek | 0 |
| San Juan (UT) | Dove Creek | 0 |
| Gunnison | Gunnison Basin | 0 |
| Saguache | Gunnison Basin | 0 |
| Mesa | Piñon Mesa | 46 |
| Grand (UT) | Piñon Mesa | 93 |
| San Miguel | San Miguel | 0 |
